# Growth of, and diffusion in, olivine in ultra-fast ascending basalt magmas from Shiveluch volcano

**DOI:** 10.1038/s41598-018-30133-1

**Published:** 2018-08-06

**Authors:** Boris Gordeychik, Tatiana Churikova, Andreas Kronz, Caren Sundermeyer, Alexander Simakin, Gerhard Wörner

**Affiliations:** 1 Institute of Experimental Mineralogy, Russian Academy of Sciences, Chernogolovka, 142432 Russia; 2 Geowissenschaftliches Zentrum Göttingen, Abteilung Geochemie, Universität Göttingen, Göttingen, 37077 Germany; 3 Institute of Volcanology and Seismology, Far East Branch, Russian Academy of Sciences, Petropavlovsk-Kamchatsky, 683006 Russia

## Abstract

Complex core-rim zoning of Mg-Fe-Ni-Ca-Cr-Al-P in high-Mg olivine crystals from a tuff ring of Shiveluch volcano, Kamchatka, enables reconstruction of the entire olivine crystallization history from mantle conditions to eruption. Bell-shaped Fo_86–92_ and Ni profiles in crystal cores were formed by diffusion after mixing with evolved magma. Diffusion proceeded to the centres of crystals and completely equilibrated Fo and Ni in some crystals. Diffusion times extracted from Fo and Ni core profiles range from 100 to 2000 days. During subsequent mixing with mafic mantle-equilibrated melt, the cores were partially dissolved and overgrown by Fo_90_ olivine. Times extracted from Fo and Ni diffusion profiles across the resorption interface between the core and its overgrowth range within 1–10 days, which corresponds to the time of magma ascent to the surface. The overgrowth shows identical smooth Fo-Ni decreasing zoning patterns for all crystals towards the margin, indicating that all crystals shared the same growth history after last mixing event prior to eruption. At the same time, Ca, and to an even greater extent Cr, Al, and P have oscillatory growth patterns in the crystals overgrowth. Our data show that magma ascent can be extremely short during maar/tuff ring eruption.

## Introduction

We have a good understanding of the principal processes of compositional differentiation and mixing of magmas prior to their eruption. However, the duration of these magmatic processes preceding eruption, i.e. differentiation, storage, mixing, and rejuvenation of melts from crystal mush, as well as amalgamation of distinct magma batches, are yet to be understood^1–7^. Also, our understanding of how fast mantle-derived magmas transit through the crust to surface is limited. Diffusion speedometry is one method to extract residence times of zoned crystals at given P-T-*f*O_2_ conditions, i.e. following recharge and mixing processes. Zoning in olivine has the potential to directly link recharge by new input of mafic melts into resident magmas, which is an eruption trigger, and ascent times from deep magma sources^1,5,8^. Many studies of zoned minerals focus on olivine and the shallow parts of magma systems in an attempt to find a link with such processes as degassing and seismic events before the eruption^9–11^. Olivine hosts a range of elements, such as Li, Al, Ca, Cr, Mn, Co, Ni, which have different diffusion coefficients. Information about zoning of multiple elements in one crystal offers the possibility of simultaneous modelling of diffusion^1,8,12–16^. Diffusion of Mg-Fe and Ni in olivine is relatively fast and therefore different stages of ascent, crystallization and mixing of mantle-derived magmas are generally obliterated during the extended history of olivine crystals. Based on Ni diffusion modeling of olivine crystals from Irazú volcano, Costa Rica, it was suggested that magmas can rise from mantle depth during a period ranging from a hundred days to a few years prior to eruption^5^. Even such short periods were sufficiently long to erase any Fo zoning in olivine. Thus, the earlier record of the deeper history of olivine crystals is difficult to constrain.

We document olivine crystals from a basaltic maar deposit at Shiveluch volcano (Kamchatka) that preserve rich records of the full history of growth and diffusion from the deep mantle source of the magmas to the Earth’s surface. Fo_92_ cores were formed from primary magmas and suffered from diffusional exchange to various extents in evolving host magmas. Recharge caused partial dissolution and renewed growth of Fo_90_ olivine and resulted in the formation of identical overgrowths on a variety of olivine cores. Subsequently, a next stage of diffusion smoothed out the compositional interface between cores and overgrowth. The overgrowths have distinct growth bands for slow diffusing Cr, Al, P, despite smooth Fo-Ni zoning. Based on Fo and Ni diffusion, we demonstrate that these olivine crystals had been growing at mantle depths for about 100 to 2000 days before their final ascent to the surface in only 1–10 days.

## Shiveluch Volcano, Kamchatka: Geology and Sampling

Kamchatka is one of the most active magmatic arcs in the world with abundant mafic lavas that contain high-Mg olivine phenocrysts. We studied here high-Mg middle-K basalts from a phreatomagmatic maar deposit in order to constrain the residence and ascent times for basalts feeding one of the most active volcanoes in Kamchatka – Shiveluch^17^, located near the northern edge of the subducting Pacific Plate^18^. The volcano is located about 90 km above the slab that descends at an angle of 35° (e.g.^19,20^). Petrological and geophysical studies suggest that a slab window beneath Shiveluch was formed by a transform fault between the Pacific and Bering Plates^21–23^ and the slab edge became heated and partially melts to import a slab-melt signature to resultant magmatism^18^. The abundance of peridotite xenoliths^24^ indicates that the magma system is fed by fast-ascending mantle-derived magmas. Shiveluch volcano erupted mainly high-Mg andesites^18,25,26^ during Holocene times, however, previous tephrochronological studies^25,27^ described two high-Mg tephra layers dated at 3600 yr BP and 7600 yr BP, respectively (^14^C dating). Our samples were derived from deposits of a phreatomagmatic maar that was recently discovered at the SW end of the Baidarny ridge^28^ and is likely the source of the 7600 yr BP tephra layer^29^. Sample SHIV-08–05 is a weakly-vesiculated ol-cpx-pl basaltic andesite bomb and sample SHIV-08-07 was collected from fine-grained scoria of the phreatomagmatic tephra deposit. Euhedral olivine grains can reach up to 8 mm in size with inclusions of chromium spinel up to 0.05 mm. These basaltic andesites have SiO_2_ = 52–54 wt.%, MgO = 8–8.5 wt.%, and K_2_O = 0.85–0.95 wt.%^28^. The composition of different mineral phases (olivine, spinel, clinopyroxene, plagioclase) in our samples can be found in Supplementary Materials (SM) Tables SM2-A, SM3-A, and SM3-B.

## Compositional Profiles and Zoning Types

**Figure 1:**
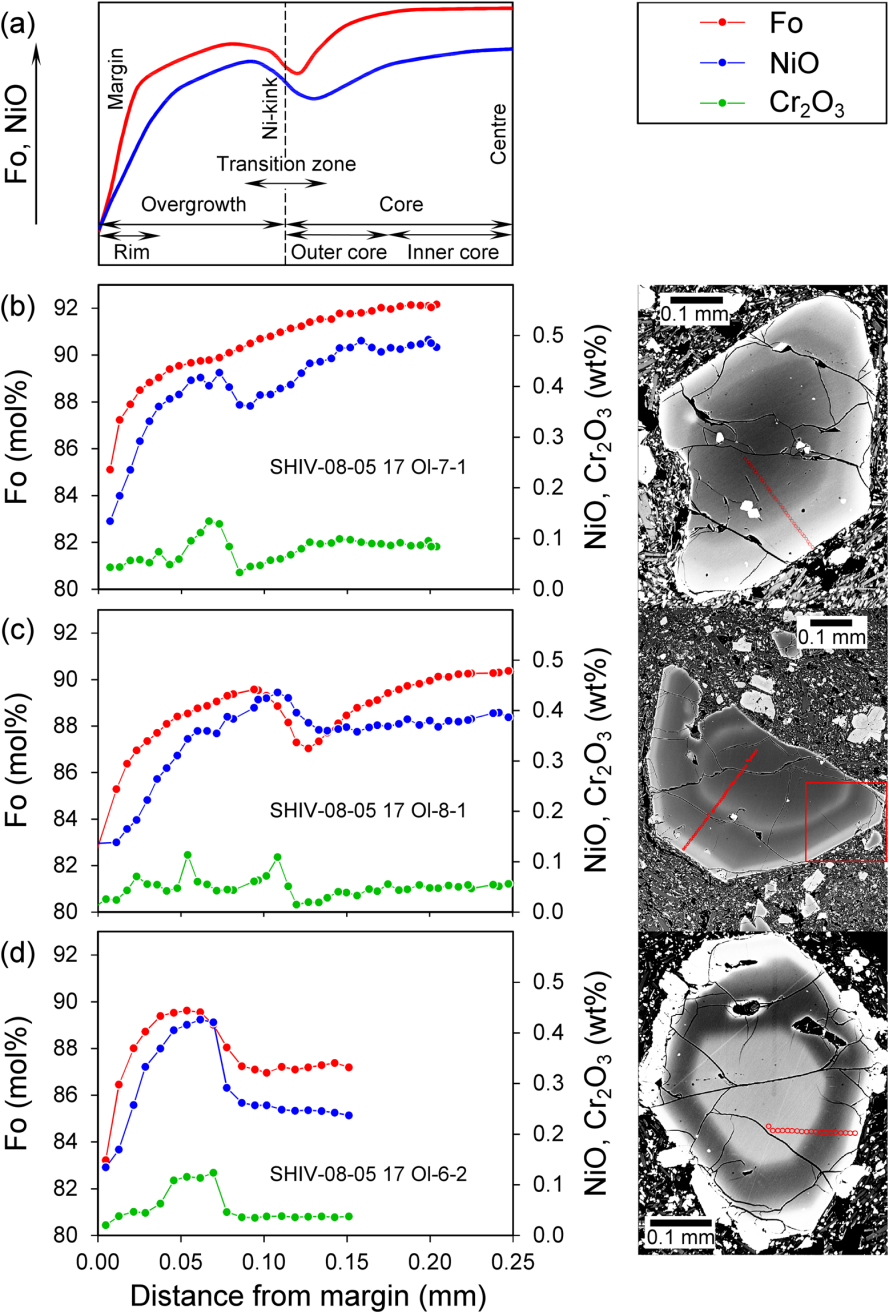
(**a**) Schematic representation of core-rim zoning defining the descriptive terms used here. (**b**–**d**) Fo, NiO and Cr_2_O_3_ distributions in representative olivine crystals and their BSE images from high-Mg middle-K Shiveluch basalt. Olivine crystals show variable zoning patterns: (**b**) – normal, (**d**) – complex reverse, and (**c**) – complex repetitive. The profiles are marked by red dots on the BSE-images. All profiles were measured from the margin of the crystal to the most central part. Forsterite values (red) are shown on the left axis, NiO (blue) and Cr_2_O_3_ (green) values are shown on the right axis. Red square in (**c**) denotes detail shown in Fig. 4.

**Figure 2:**
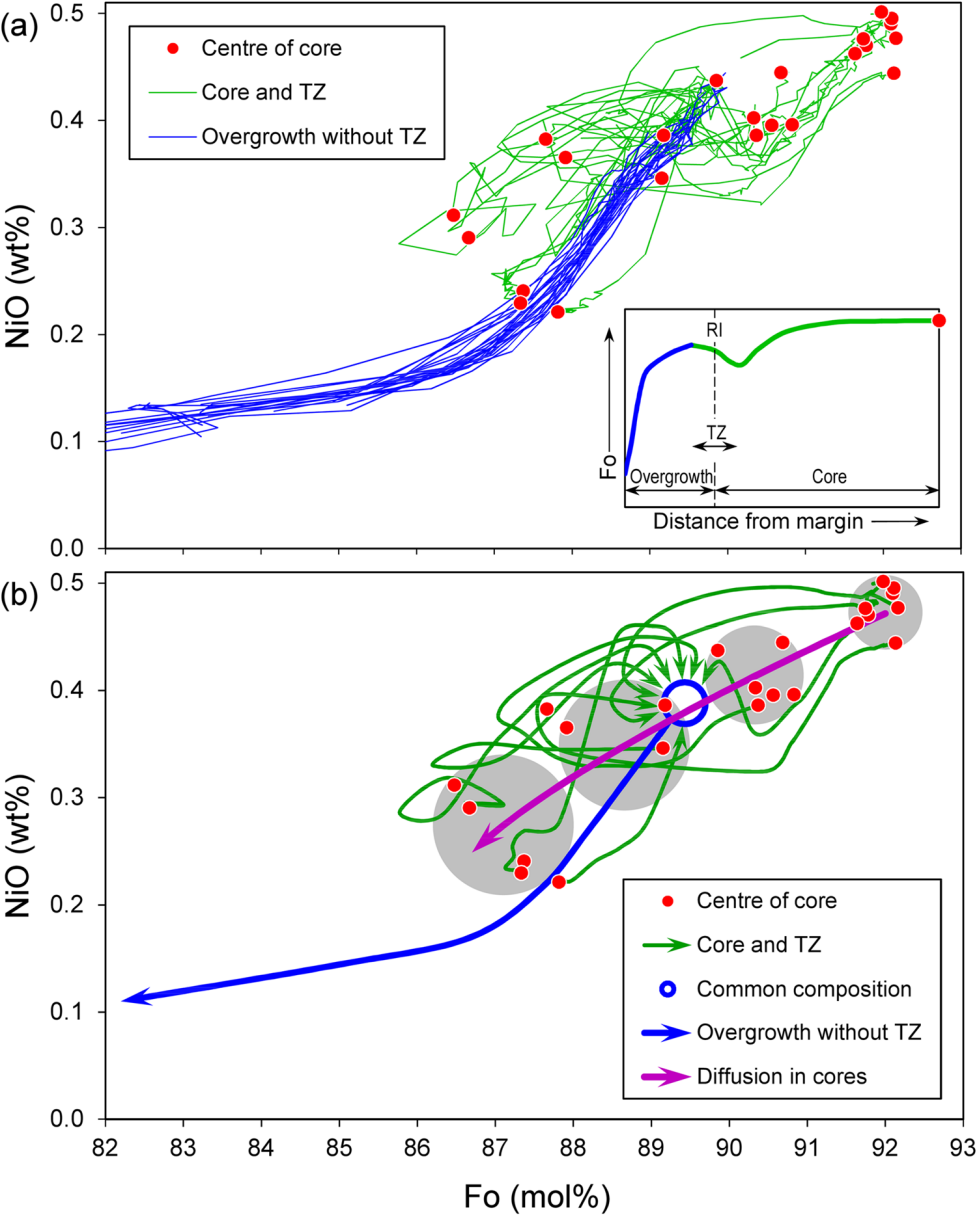
Fo-Ni variations along compositional profiles: (**a**) – measured olivine crystals and (**b**) – schematic representation emphasizing growth and diffusion histories. Measurements show distinct core compositions (red dots) and complex and irregular trajectories from the centres to transition zones (green lines) all coming to a common composition (blue circle). Fo-Ni depletion trend starts from this common composition and compositional zoning towards the crystal margins is similar for all crystals (blue lines). RI – resorption interface, TZ – transition zone.

**Figure 3:**
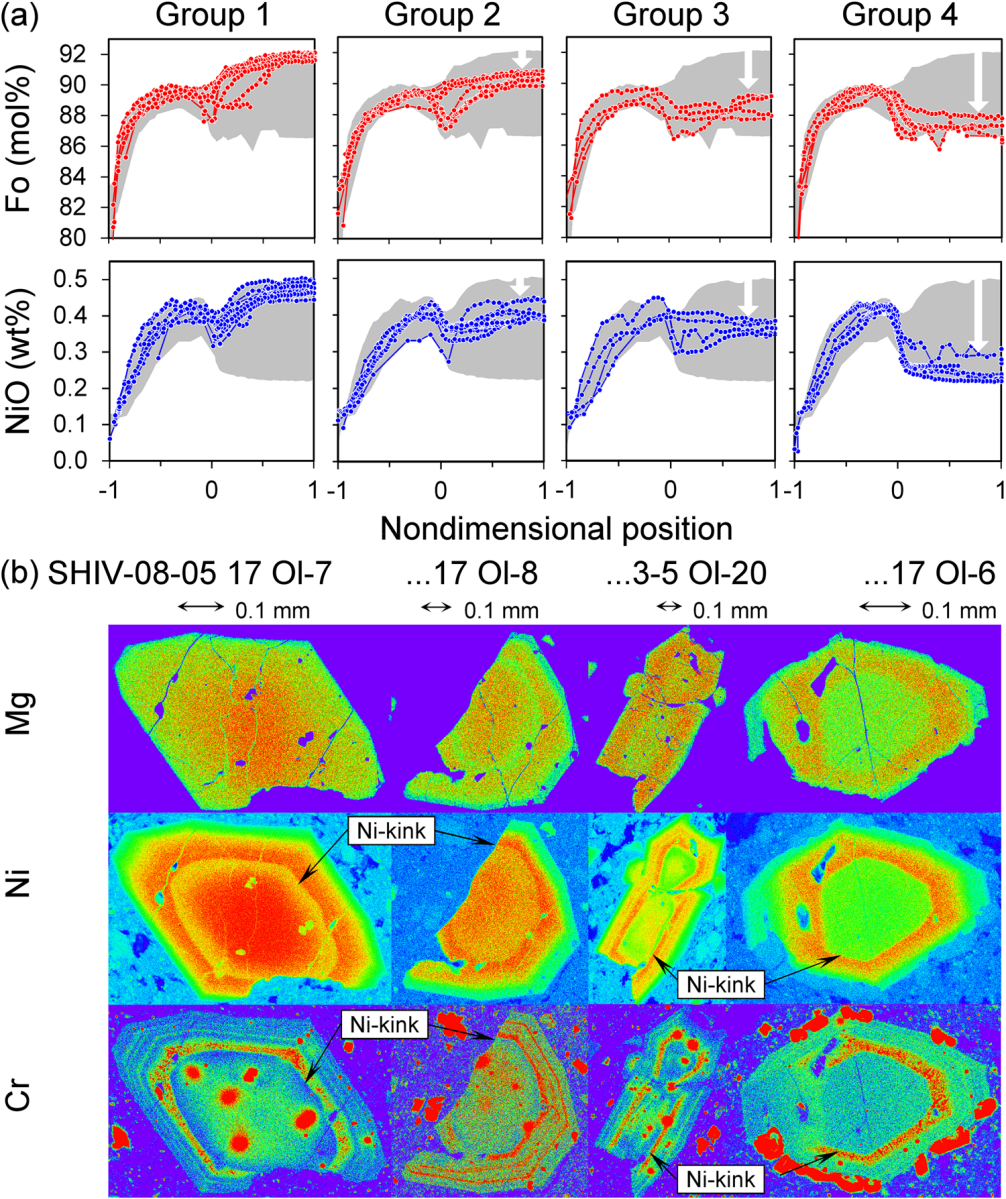
(**a**) Olivine crystals with Fo and Ni zoning are divided into four groups. Spatial axes along the profiles are in dimensionless coordinates: the margin, the Ni-kink, and centre of each crystal are allocated coordinates of −1, 0, and 1, respectively. Grey fields show all data for reference. The shapes of gradients in the cores change from bell-shaped to flat from group 1 to 4. Forsterite and nickel contents in the centre of the cores decrease with increasing diffusive equilibration to a more evolved melt (“advanced core diffusion”, white arrows) (**b**) Representative compositional maps of crystals for four different groups of cores for elements of different diffusivities. The spectrum colors from red to violet correspond to element concentration from maximum to minimum. Note the smooth compositional variations for Fe-Mg distributions. Ni shows more structure and clearly defines the dissolution interface (Ni-kink) between outer core and subsequent overgrowth. Cr is an element with slow diffusivity and defines the dissolution boundary between the core and the overgrowth more clearly than Fe, Mg, and Ni. Cr also has retained delicate oscillatory growth zoning in the overgrowth on the cores. The high-chromium inclusions in olivine are chromite.

Compositional zoning in high-Mg olivine is documented by 27 profiles across 19 grains for elements with different diffusivities (Mg, Fe, Ni, Ca, Cr, Al, P; Figs 1–3, Supplement SM2, and Tables SM2-A, SM2-C). Olivine crystals show distinctly different Mg-Fe distributions: normal zoning (Fig. 1b), complex reverse zoning (Fig. 1d), and complex repetitive zoning (Fig. 1c), indicating different growth-diffusion histories. An example of an unusual Fo-Ni zoning in SHIV-08-05 17 Ol-8-2 profile is analysed in detail in Supplement SM2.1.

We define different zones (Fig. 1a) to aid the data presentation and discussion. The ***core*** includes the area from the ***centre*** of a crystal to a prominent resorption surface. This smoothly curved interface with abundant melt/fluid inclusions occur in all crystals (Fig. 1) and all element maps, indicative of partial dissolution prior to further growth. The analysis shows that the resorption interface is also represented in the profiles as a point of NiO inflection, referred to as the ***Ni-kink*** here. Cores of olivine grains have bell-shaped (Fig. 1b,c) to flat (Fig. 1d) forsterite and nickel distributions. Fo varies from 92 to 86 mol.% in the core and we distinguish between the ***inner core*** with minor Fo-Ni variations and the ***outer core*** with steeper compositional gradients. An ***overgrowth*** is formed on the cores after the resorption event and extends from the Ni-kink to the crystal ***margin***. We term the area between core and overgrowth, i.e. around of Ni-kink, the ***transition zone***. Points of inflection at the dissolution interface also occur in the Fo-profiles, but are not as clearly expressed as for NiO. The transition zones are variable in width in different grains but do not exceed 0.02 mm for NiO and 0.04 mm for Fo. The ***rim*** is defined as an outer part of the overgrowth which is characterized by a sharp drop in Ni and Fo close to the margin of the crystal (Fig. 1).

The Fo-Ni diagram (Fig. 2a) shows individual Fo-Ni profiles and their schematic representation (Fig. 2b). To understand the continuous evolution of olivine compositions, dissolution, growth and subsequent diffusion, we distinguish four groups, from a high-Mg-high-Ni group 1 to the low-Mg-low-Ni group 4 (Fig. 2b). Centres of cores (red points in Fig. 2) and their corresponding groups (grey circles in Fig. 2b) form a gentle concave downwards trend in the Fo-Ni diagram. The parts of profiles from the centre across the cores and transition zones are represented by green lines in Fig. 2a and show irregular tangled trajectories, depicted schematically in Fig. 2b. These compositional trajectories originate at distinct core compositions but all meet at a common composition of about Fo_89_ and NiO 0.4 wt.%. From this point, the compositions in the overgrowths and rims (blue lines in Fig. 2a) unite and all follow identical paths of smoothly decreasing Fo and NiO (represented by a single blue line in in Fig. 2b). This means that the last stage of evolution in terms of Fo and Ni was similar for all crystals.

To better compare profiles for many olivine crystals of different sizes, we used dimensionless spatial coordinates in Fig. 3a. The margin, the Ni-kink, and centre of each crystal are allocated coordinates of −1, 0, and 1, respectively. The transformations are linear in both intervals inside and outside of the Ni-kink. Such normalized profiles of Fo and NiO (Fig. 3a) for every group of olivine allow us to more clearly define distinct cores and transition zones, as well as common overgrowths and their rims. The shapes of the core Fo and Ni profiles vary continuously from bell-shaped to flat from group 1 to 4; Fo and Ni level differences increases in the transition zone from group 1 to 4, while zoning of the Fo and Ni in the overgrowth beyond the transition zone is similar in all groups.

Group 1 cores have well-defined bell-shaped distributions for forsterite and nickel (Fig. 3). These zoning patterns are reminiscent of diffusion profiles. All inner cores have identical high Fo_91.6–92.2_ and NiO = 0.45–0.5 wt%. Their rims indicate equilibrium with less mafic and lower-Ni melt. These cores with bell-shaped Fo and Ni distributions contrast strongly with group 4 cores, which have low and flat Fo and NiO distributions inside the core (Fo_86.5–87.8_, NiO = 0.22–0.31 wt.%). Groups 2 and 3 are intermediate and their cores are successively lower in Fo and NiO (group 2: Fo_89.9–90.8_, NiO = 0.39–0.45 wt.%; group 3: Fo_87.7–89.2_, NiO = 0.34–0.39 wt.%) and their zoning patterns become flat. Even though the Fo contents and zoning patterns of these various cores (1–4) are different, the trace element concentrations of slowly-diffusing elements (Ca, Al, P) are similar, indicating a common origin.

**Figure 4:**
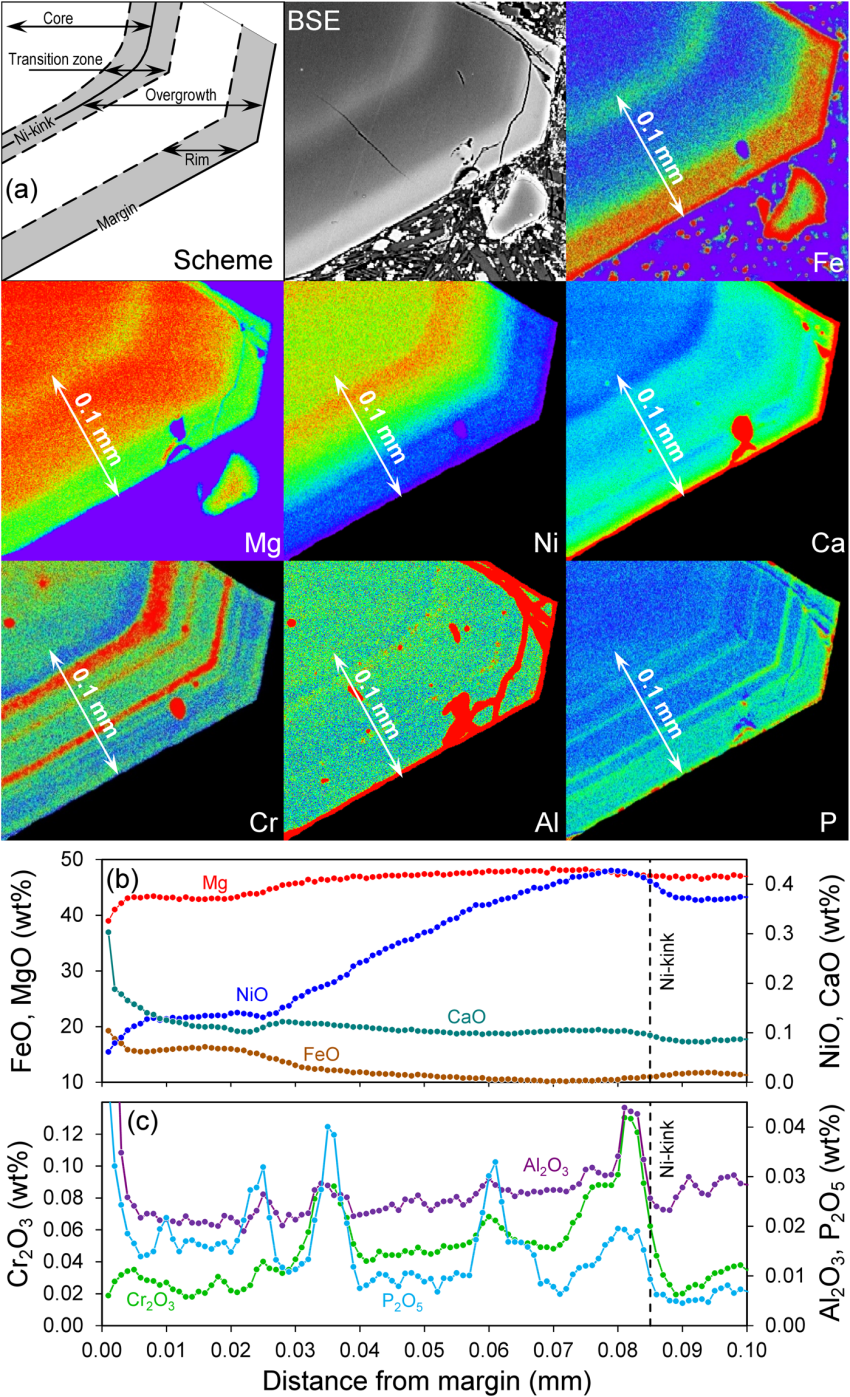
(**a**) Fe-, Mg-, Ni-, Ca-, Cr-, Al-, and P-distribution maps in the olivine crystal showing distinct growth zones. Here we show only a small part of crystal SHIV-08-05 17 Ol-8 that is marked by a red frame in Fig. 1c. The spectrum of colors from red to violet corresponds to element concentration from maximum to minimum. Many other crystals that we analyzed have similar zoning patterns. (**b**,**c**) – detailed profile with steps of 0.001 mm, marked by white arrows on (**a**), shows variable widths of the high-concentration zones, which depend on their relative diffusivities (P < Al < Cr < Ca < Fe-Mg). For example, the half-widths of Al peaks are only 2–3 microns. Ca and Ni profiles are increasingly smoother and Fe-Mg profiles show almost equilibrated profiles across peaks for P, Al, Cr. This clearly indicates that the overgrowths of the olivine crystals were initially zoned in all these elements during crystallization but subsequent diffusion has partially erased Mg/Fe and, less so, Ni zoning patterns.

The Fo-Ni composition of different inner cores in Fig. 2 shows a distinctly concave trend that is not consistent with fractional crystallization. By contrast, the overgrowth shows convex downwards Fo-Ni trends, which is consistent with fractional crystallization^5,30–33^. These observations indicate that (1) the cores had a similar origin but different history that has affected the Fe-Mg distribution, (2) the cores and their overgrowths were formed by distinct processes from different magmas and (3) after dissolution of the cores the crystals continued to grow from increasingly differentiated magma. While Fo and Ni smoothly decrease in the overgrowth, all slow diffusing elements (Ca, Cr, Al, P) show sharp oscillatory growth patterns (Figs 3b, 4).

## P-T-*f*O_2_ Conditions of Olivine Crystallization

Pressures determined from cpx-melt equilibria range from 6 to 10 kbar (18–30 km) consistent with the results of cpx-only barometry (6 kbar)^34^. These are in good agreement with previous estimates^35^ (7–9 kbar). Olivines with Fo_86–92_ were formed at similar or even greater depths. The rationale for this is: (1) pyroxene crystals were formed after olivine and are invariably smaller grading into cpx of the fine-grained matrix, (2) pyroxene Mg# varies from 87 to 68 (Table SM3-B) indicating crystallization from more evolved melt than that of the olivines, and (3) pyroxene lacks overgrowths on resorbed cores unlike the more complex zoning observed in the olivine crystals.

Temperature estimates are based on Al-in-olivine thermometry^36^, which utilizes the Al-Cr distribution between olivine and spinel. We used the composition of the local spinel inclusion adjacent to the host olivine for every zone (Fig. 1a). Temperatures for the inner cores of olivines range from 1230 to 1260 °C and are lower in outer cores (1170–1190 °C; Table SM3-C). Temperatures vary from 1150–1220 °C in the transition zone to 1160–1200 °C and 1130–1155 °C in the overgrowth towards the rim, respectively. Pre-eruption temperatures of around 1100 °C in Shiveluch lavas^35^ are similar to the temperatures estimated from the rim of our olivine crystals. The relatively high pressures and temperatures suggest that the cores and overgrowth of the olivine crystals were formed under mantle conditions.

Numerical simulation of the temperature distribution in the mantle wedge under Shiveluch^37^ provide indirect evidence of the maximum depth of origin for the olivine cores. Temperatures that we estimated for olivine core crystallization are 1130–1260 °C and such temperatures are predicted at a depth of 55–80 km.

Oxygen fugacity is ΔQFM = +1.04 ± 0.26 at a pressure of 6 kbar and ΔQFM = +0.90 ± 0.26 at 10 kbar (Table SM3-A), both being somewhat lower than previously estimated^35^ for similar lavas ΔQFM = +1.8 ± 0.15.

## Discussion: Growth and Diffusion History

The olivine crystals record two distinct stages of crystallization for core and overgrowth, which are separated by a dissolution interface. With respect to diffusion in the core, we distinguish two stages (Fig. 3a): (1) outer core diffusion that does not reach the centres of the crystals, preserving initial Fo and Ni values in their inner cores (group 1); (2) advanced core diffusion smoothed out the gradients and also decreased the Fo and Ni in the centres of the cores forming olivine groups 2–3 with partial and olivine group 4 with complete equilibrium with a more evolved melt. A third stage of diffusion (3) occurs across the dissolution interface between core and its overgrowth that formed after recharge by a more mafic magma. A last diffusion stage (4) affected the overgrowth towards the crystal margin. We will now discuss these four diffusion stages and try to assess time scales by modeling their gradients.

*Outer core diffusion* has not affected the maximum values of Fo and NiO in the inner core. Initial compositions of the flat core^38–40^ Fo_core_ = 92 mol.% and NiO_core_ = 0.5 wt.% indicate that these crystals were formed in a mantle-derived magma^41^. Subsequently, due to mixing with more evolved melt, the composition of the outer cores equilibrated with a new melt to values of Fo_dm_ and NiO_dm_. Here “dm” refers to the original (now dissolved) margin of the crystals (now cores) that have been modified by dissolution and overgrowth. The analytical solutions for one-dimensional diffusion can be formulated for both, Fo and Ni and describe the profiles from the margin with coordinate x_dm_ into the core:

1 $$Fo=F{o}_{dm}+(F{o}_{core}-F{o}_{dm})Erf(\frac{1}{2\sqrt{{D}_{Fo}}}\cdot \frac{(x-{x}_{dm})}{\sqrt{t}}),$$

2 $$NiO=Ni{O}_{dm}+(Ni{O}_{core}-Ni{O}_{dm})Erf(\frac{1}{2\sqrt{{D}_{Ni}}}\cdot \frac{(x-{x}_{dm})}{\sqrt{t}}).$$

Where: Erf – error function, t – time, x – spatial along the diffusion profile, D_Fo_ and D_Ni_ – the diffusion coefficients for Fo and Ni, respectively. The values with the index “core” refer to the centre of a crystal.

Elimination of (x-x_dm_)/t^1/2^ from the analytical solutions gives equation 3 linking Fo and Ni variations through the inverse error function Erf^−1^ along the diffusive profiles independent of diffusion time and x_dm_ position:

3 $$Er{f}^{-1}(\frac{NiO-Ni{O}_{dm}}{Ni{O}_{core}-Ni{O}_{dm}})=\sqrt{\frac{{D}_{Fo}}{{D}_{Ni}}}Er{f}^{-1}(\frac{Fo-F{o}_{dm}}{F{o}_{core}-F{o}_{dm}}).$$

**Figure 5:**
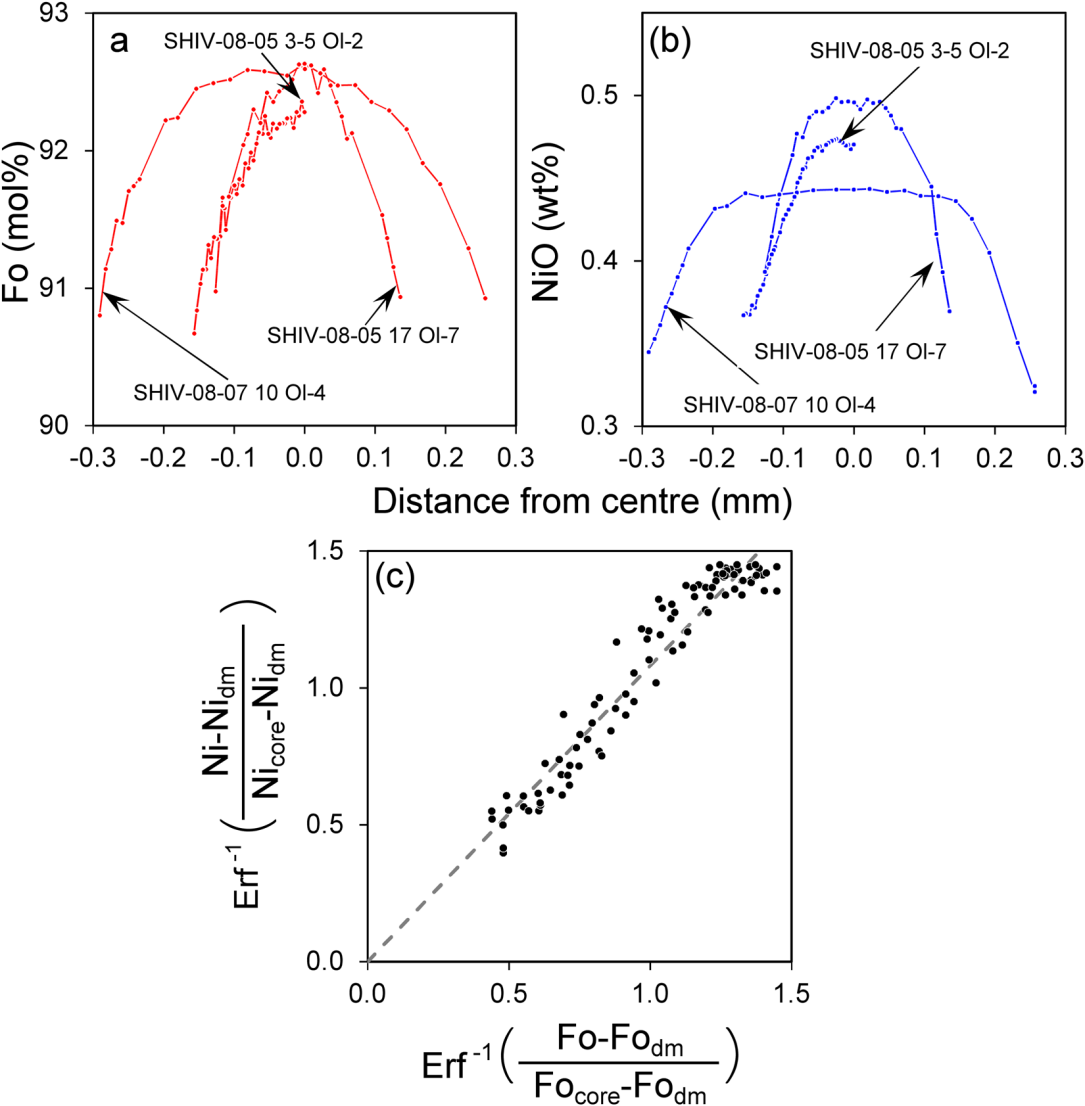
Fo (**a**) and NiO (**b**) profiles across Mg-rich olivine cores from group 1 show diffusion after the mixing event with a more evolved melt (which is in equilibrium with Fo_margin_ = 88.7 mol.% and NiO_margin_ = 0.23 wt.%). Diffusion only affected the outer cores, the inner cores remained at maximum values of about Fo_92_. (**с**) The plot shows the correlation between inverse error function for Fo-values and inverse error function for Ni-values. The slope of the dashed line shows the D_Ni_/D_Fo_ ratio of 0.86 for the outer core diffusion.

This simple formula represents diffusive Ni and Fo co-variations by a linear relation where the slope only depends on the relative values of the diffusion coefficients. Figure 5 shows three examples from outer core diffusion profiles. Figure 5c with Fo_dm_ = 88.7 and NiO_dm_ = 0.23 wt.% documents a linear correlation between left and right sides of Eq. 3 for three crystals which directly confirms the diffusive nature of the compositional gradients. From the slope of the regression line, the relative diffusions coefficients are determined to be D_Ni_/D_Fo_ = 0.86.

**Figure 6:**
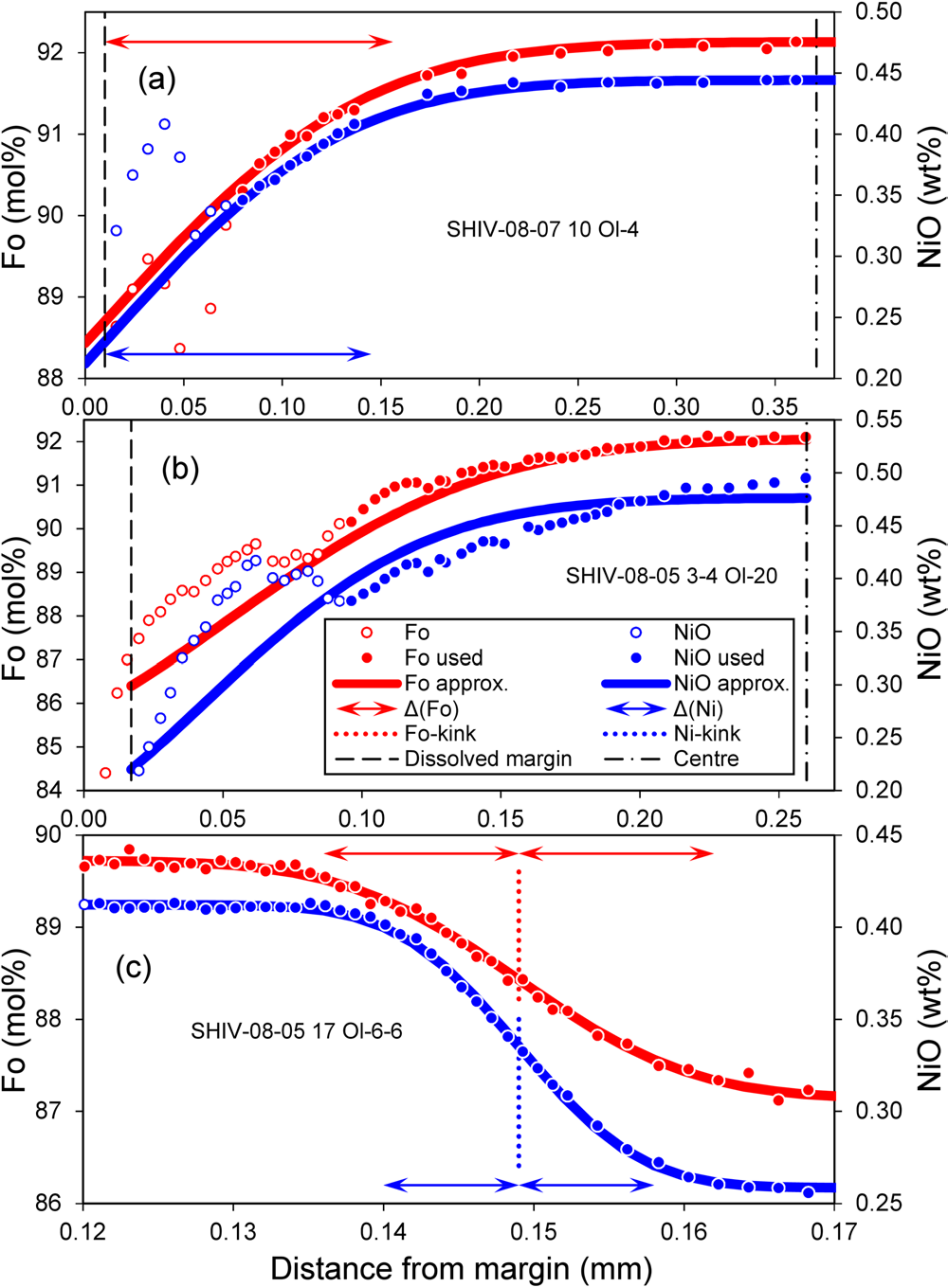
Three representative profiles with model approximations of the observed data used for the diffusion time estimates: (**a**) – outer core diffusion, (**b**) – advanced core diffusion, and (**c**) – diffusion between core and overgrowth. Fo profiles are shown in red (left axis), and NiO profiles shown in blue refer to the axis on the right. Only data points with filled circles were considered in the modeling. The solid lines show the calculated model result based on an analytical approximation (described in Methods section and Supplements SM4-SM6). Red and blue arrows are the widths of diffusion zones Δ_Fo_ and Δ_Ni_, respectively. Dashed lines are the calculated positions of the dissolved margin of the cores before their resorption. Dash-dotted lines are the positions of crystal’s centre. Red and blue dotted lines are the positions of the resorption interface across which diffusion occurs between core and overgrowth for Fo and Ni, respectively.

**Figure 7:**
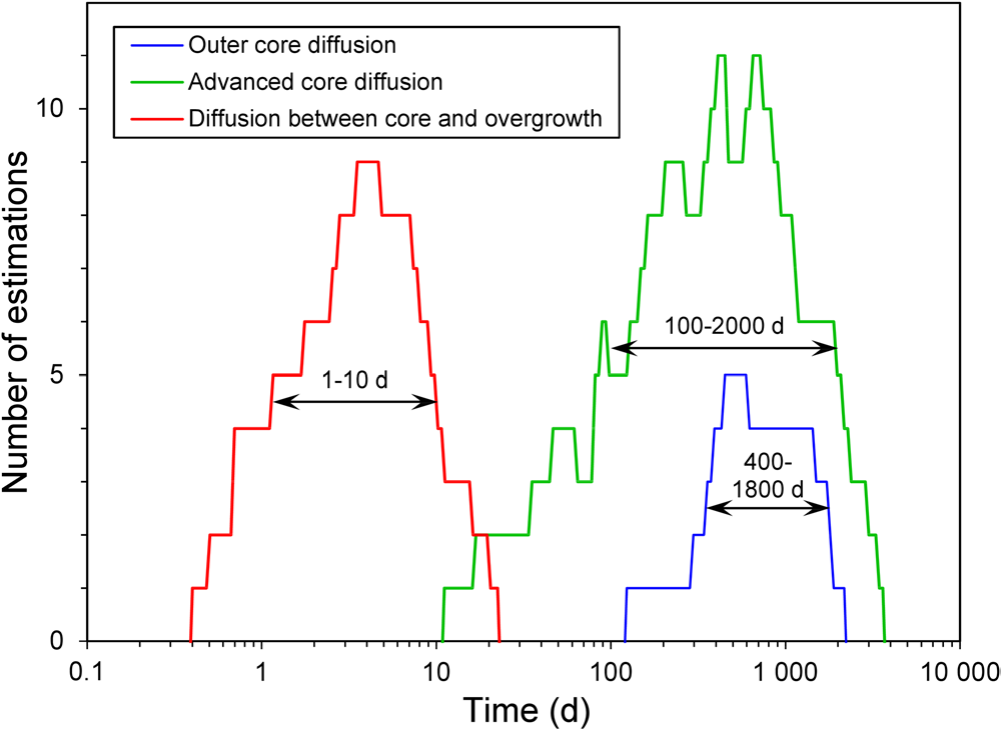
Results of individual diffusion time calculations for many measured gradients of the different diffusion stages in the form of a frequency diagram: for outer core diffusion (blue line), advanced core diffusion (green line) and diffusion across the resorption boundary (red line). Ranges for diffusion time values are defined by the half-width of the distributions.

We modeled diffusion times as explained in Methods section and Supplement SM4. Table SM4-A contains the results for 5 profiles on 3 grains from group 1 olivine cores, an example of our modeling can be found in Fig. 6a. The diffusion time estimated for outer core diffusion ranges from 400 to 1800 days (Fig. 7, blue line).

*Advanced core diffusion* was the next stage of outer core diffusion. Advanced core diffusion not only smoothed the gradients, but also decreased the Fo and Ni in the inner cores. This suggests diffusive equilibration of olivine cores with a more evolved melt that was incomplete in groups 2 and 3 but complete in group 4 (Fig. 3).

The centres of the four groups of olivine cores (red dots in Fig. 2) exhibit a low-sloping, scattered Fo-Ni trend with a concave downwards shape that contrasts with typical convex fractionation crystallization trends. Such a concave trend has not been previously described for olivines from arc rocks. To interpret this finding we consider the problem of Fo and Ni decrease by diffusion with respect to different diffusion coefficients for D_Fo_ and D_Ni_. Fo_core_ and Ni_core_ are the initial values in the crystal at time 0, Fo_dm_ and Ni_dm_ are compositions for olivine in equilibrium with more evolved surrounding melt on the dissolved margin of the crystal. By analogy with heat conduction, it is possible (e.g.^42^) to describe chemical diffusion and the change of Fo and NiO in the crystals as a function of time using Newton’s cooling law^43^:

4 $$Fo=F{o}_{dm}+(F{o}_{core}-F{o}_{dm})\cdot \exp (-t/{\tau }_{Fo}),$$

5 $$NiO=Ni{O}_{dm}+(Ni{O}_{core}-Ni{O}_{dm})\cdot \exp (-t/{\tau }_{Ni}).$$

Here τ is relaxation time, which depends on the size and form of the crystals and is inversely proportional to the diffusion coefficient. By cancelling out time and using the relation between relaxation times and diffusion coefficients, the dependency of Fo and NiO on time (Eqs 4–5) can be reduced to a function that describes the dependence of NiO from Fo as expressed in Eq. 6. This equation gives the dependence of Ni from Fo for a high-Mg and high-Ni crystal that was exposed to a low-Mg and low-Ni melt:

6 $$NiO=Ni{O}_{dm}+(Ni{O}_{core}-Ni{O}_{dm})\cdot {(\frac{Fo-F{o}_{dm}}{F{o}_{core}-F{o}_{dm}})}^{\frac{{D}_{Ni}}{{D}_{Fo}}}.$$

Using the values Fo_dm_=86.4 mol.% and NiO_dm_ = 0.22 wt.% from group 4 olivine, the D_Ni_/D_Fo_ relation was determined to be 0.74 by least squares fitting of the observed core compositions, which are represented by red points in Fig. 2b. The dependence of Fo is quantitatively represented in Fig. 2b by the violet line. In our case D_Ni_/D_Fo_ < 1 therefore the violet line has a concave curvature, which is distinctly opposite to the convex trend for fractional crystallization represented by the blue line in Fig. 2b.

However, Newton’s cooling law and its diffusion analogy have a number of limitations: (1) the crystal is treated as a point object, (2) the law is not applicable to partial diffusion that does not reach the centre of the core and so does not describe the complete process from its starting point in time, and, finally (3) anisotropic properties of the crystal are not considered.

On the other hand, these limitations do not affect the curvature of the modeled diffusion trend. The problem regarding component concentration in a diffusion sphere with a finite radius at any time is accurately solved by equation 6.18 from ref.^44^. Calculations using this complex formula with an exponential series instead of Newton’s cooling law (Eqs 4–5) and the detailed numerical simulation of anisotropic diffusion in crystals of various shapes and size also all yield a concave shape for the Ni-Fo relation at D_Ni_ < D_Fo_. The details of these analyses, however, are beyond the scope of this paper.

The use of Newton’s cooling law as an analogue for diffusion provides a simple analytical explanation for the concave trend for high-Mg high-Ni olivines undergoing diffusion and re-equilibration after being exposed to a low-Mg, low-Ni melt at D_Ni_ < D_Fo_ (violet line, Fig. 2b). In other words, the concave trend shows that the crystal exchanges Fo faster than Ni with the new melt as long as D_Ni_ < D_Fo_. Thus, concave trends in Ni-Fo space in olivine that run contrary to typical trends during fractional crystallization can be taken as solid evidence for diffusion.

The Methods section and Supplement SM5 document the model fits by equation 6.18 from ref.^44^ for 18 profiles on 15 grains from which diffusion times were extracted, except for the fully equilibrated cores of group 4. Examples of such model fits can be found in Fig. 6b. Based on the entire data set, we conclude that the time scale of diffusion that modified the outer cores of the crystals range from 100 to 2000 days (Fig. 7, green line). Since outer core diffusion is only a particular case of advanced core diffusion for the largest grains, both diffusion time estimates should be similar, which is indeed the case (400 to 1800 days for outer core diffusion vs. 100 to 2000 days for advanced core diffusion).

Such complexly Fo- and Ni-zoned cores of olivine crystals have not been documented before and is most likely due to fast Fo-Ni diffusivity and magmatic residence times at high temperatures during and/or after ascent are typically much longer. This observation and our modeling then implies that most mafic magmas have transfer times from source to surface that would normally be >2000 days or longer. Instead, our olivine crystals from Shiveluch must have had unusually fast ascent times, as discussed in more detail below. Another conclusion we made is that concave Fo-Ni trends reflect a diffusion process with D_Ni_ < D_Fo_ (Fig. 2b). Such concave compositional trends have previously been documented in olivine crystals from komatiites and kimberlites^45–51^ and probably indicate similar diffusion process.

*Diffusion between crystal core and overgrowth* followed after mixing with, and growth from, a more mafic higher-temperature recharge magma into the reservoir where the olivine crystals resided in a more evolved melt. The diffusion interface is clearly marked by the sharp breaks in concentrations of slowly diffusing elements (Cr, Al and/or P, Figs 1, 3, 4, Tables SM2-A, SM2-C), as well as by Ni-kink between outer cores and overgrowth (Figs 1, 3, 4, Tables SM2, SM2-C). The Fo and NiO profiles from group 4 olivines (Fig. 3a) are the best for diffusion modeling as the initial/boundary conditions allow the application of the analytical solutions.

As an alternative interpretation to the origin of the four groups of olivine cores, low-Mg olivine could also form after fractional crystallization in a more evolved melt, rather than exchange by diffusion with an evolved melt after magma mixing. Irrespective of the origin of group 4 olivine cores, a magma mixing event is clearly indicated by the resorption interface and so we can use the width of the transition zone to determine the time elapsed since the mixing event. As emphasized above, there are continuous transitions from core group 1 to group 4, which would not be expected if there had been two distinct processes and two distinct magmas that formed these cores. Therefore, we prefer the interpretation of diffusive exchange to explain the different core types.

We measured and modeled diffusion of both Fo and NiO across the resorption interface (the analytical solutions of the diffusion equations and comparison with observed gradients are described in Methods section and Supplement SM6). The width of the diffusion zones for Ni are consistently narrower than that for Fo, suggesting D_Ni_ < D_Fo_. In previous studies, such diffusion modeling was performed separately either for forsterite^38^ or for nickel^5^ and modeling of olivine profiles with simultaneous exchange Fo and NiO has only been described previously for the rims of crystals^1,52^. However, as we show in this study, such rims may be affected by both diffusion and crystal growth and comparing results for Ni and Fo may not be reasonable in this case. However, the measured data and our modeling across the resorption interface allows us, for the first time, to directly assess the contribution of each diffusion component.

The modeling of diffusion time is documented for 8 profiles on 3 grains across the transition zone for group 4 cores in Table SM6-A. An example of a model fit to the data can be found in Fig. 6c. The diffusion times range from 1 to 10 days (Fig. 7, red line). Thus, the time of diffusion between core and overgrowth is orders of magnitude shorter than the time of diffusion that had affected the cores prior to their resorption.

*Zoning and diffusion in the overgrowth* must have postdated the resorption event and at least partly the subsequent overgrowth. All profiles outside the transition zone towards the margin show the same compositional Fo-Ni trends (blue lines in Fig. 2). This and oscillatory growth bands in Cr, Al, and P (Figs 3, 4) clearly demonstrates that (1) all crystals had a common history after the mixing event and (2) this history was dominated by crystallization from the current host melt. The widths of the Fo and Ni gradients in this growth zone are significantly wider than widths across the resorption interface. This indicates that smoother gradients within the overgrowth cannot result from diffusion, because the time of diffusion within cannot be longer than for diffusion further inside the crystal. Thus, the formation of the overgrowth reflects olivine growth in a progressively evolving melt. Repeated excursions to more mafic compositions (high Cr) are inferred to reflect minor recharge events during the fractional crystallization process. However, these high Cr growth zones are also enriched in Ca, Al, and P. Slow-diffusing P can be strongly affected by slow kinetics during fast crystal growth^13,53–55^. Therefore we ascribe the narrow oscillatory growth bands to kinetic effects rather than growth from changing melt compositions. In any case, diffusion after growth has partially smoothed out variations in Mg, Fe, Ni and – less so – Ca, but not for Cr, Al and P.

## Conclusions

**Figure 8:**
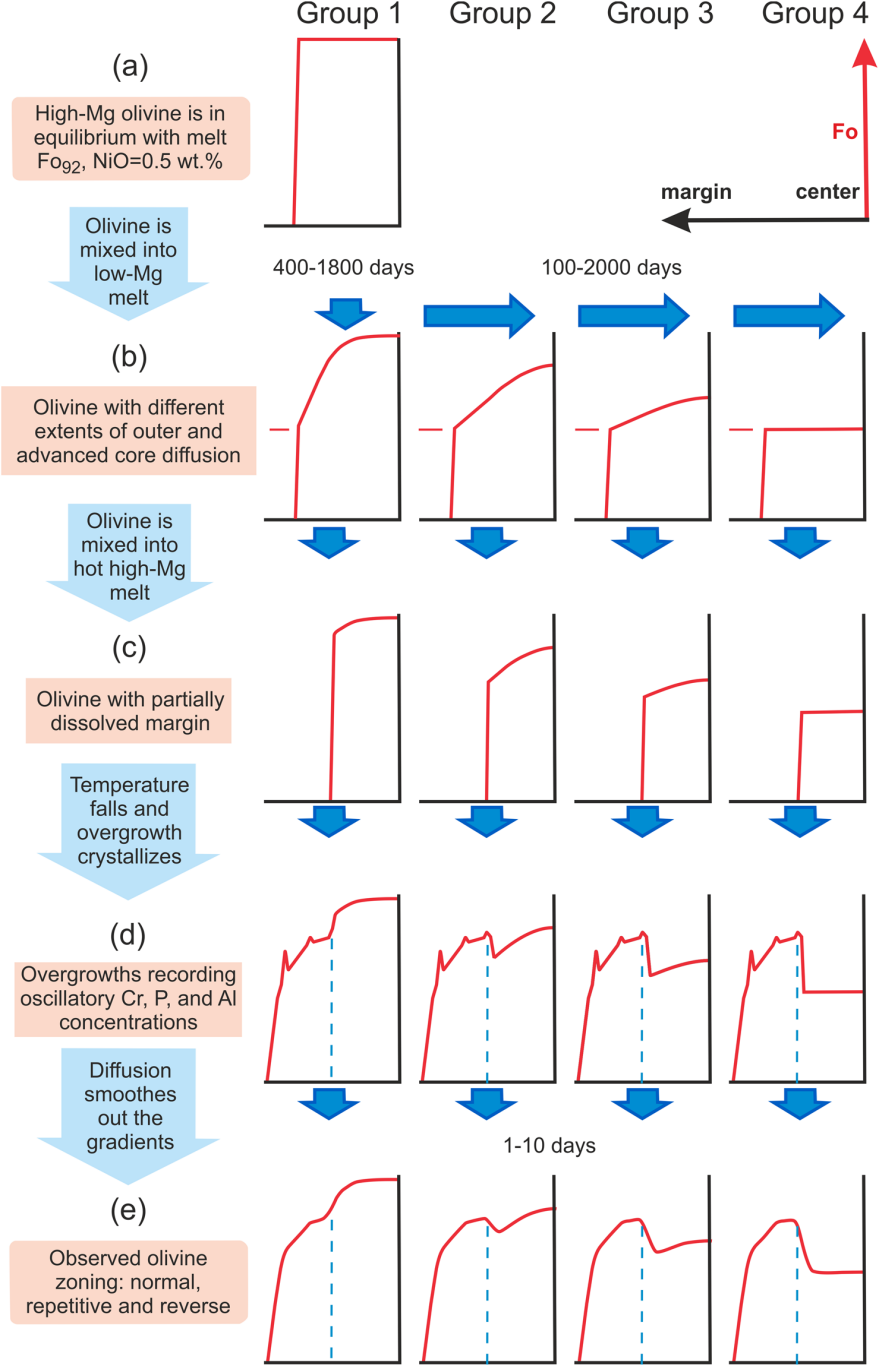
The schematic evolution of the complex growth, dissolution and diffusion history of olivine crystals from high-Mg middle-K Shiveluch basalt is depicted in simplified centre-to-margin Fo-profiles. (**a**) The high-Mg olivine with a flat Fo-distribution were formed first. (**b**) High-Mg cores were exposed to a low-Mg melt after magma mixing. As a result, olivine crystals were affected by diffusion to differing extents and decreasing Fo is observed. (**c**) Recharge with a hot high-Mg melt dissolved outer parts of the zoned olivine crystals, and (**d**) high-Mg overgrowths were formed over the different types of resorbed cores. (**e**) Finally, diffusion across the resorption interface and across the overgrowth started. Compare these final Fo-profiles to the measured profiles in Fig. 1 and Fig. 3. Blue dotted line indicates the position of the resorption interface.

Successive stages of olivine growth and diffusion history are schematically shown in Fig. 8 and described below:

1. Olivine cores were formed from a high-Mg and high-Ni melt and initially had a uniform Fo and Ni composition (Fo_92_ and NiO~0.5 wt.%; Fig. 8a).
2. These olivine crystals encountered a more evolved melt in which they diffused to different extents towards an equilibrium composition of Fo_86,4_ and NiO~0.22 wt.% at their rims (Fig. 8b). When the diffusion process did not reach the inner cores (group 1) this indicates times scales ranging from 400 to 1800 days (Fig. 7, blue line). The inner cores of olivine of groups 2 and 3 were affected by diffusion to variable degrees with diffusion times ranging from 100 to 2000 days (Fig. 7, green line). Some olivine crystals completely equilibrated with this evolved melt (group 4).
3. The next event in the history of these olivine crystals was magma mixing with a new high-Mg and high-Ni melt. Due to the high temperature of this melt, the rims of olivine cores were partly dissolved creating a prominent resorption interface between cores and their overgrowths (Fig. 8c).
4. Subsequent cooling resulted in new high-Fo and high-Ni olivine growths over the resorption interface that formed the overgrowth of the crystals (Fig. 8d). Newly grown olivine in this zone is enriched in Cr_2_O_3_ up to 0.3–0.4 wt.%. Further crystallization produced olivine down to Fo_80_ towards the rims of crystals and concentric, oscillatory and correlated growth bands of slow-diffusing elements (Cr, Al, P; Fig. 4) that most likely formed by kinetic effects during fast crystal growth.
5. While Cr, Al, and P show sharp growth bands in the overgrowth, Fo and NiO variations were partly smoothed out by diffusion at the same time during which diffusion modified the resorption interface (Fig. 8e). The duration of this last growth and diffusion stage is only 1–10 days (Fig. 7, red line). This suggests a very short time between the last recharge event (core resorption) and surface eruption.

Thus, high-Mg high-Ni overgrowths on the cores were formed only a few days after the last mixing event and this mixing event likely triggered the eruption. The composition of recharging melt at the resorption interface has Mg# = 64–65^28^, i.e. it is close to a mantle-derived melt and from this we conclude that mixing took place close to mantle levels which is in accord with the P-T-fO_2_ conditions of olivine crystallization (18–30 km). Therefore, ascent from such depths should have taken place within 1 to 10 days, implying ascent rates of 80 to 1200 m/h. By contrast, the formation of the cores, their storage and magma mixing and diffusion must have all occurred at mantle levels around 100 to 2000 days before eruption.

Fast ascent may be more frequent than previously thought because slow cooling during the late stages of ascent and cooling in lava flows or recharge into larger shallow magma reservoirs within volcanic edifices, generally erases zoning records like those described here. One possibility for preserving records of fast ascent of mafic magmas from great depth is the nature of the maar eruption which has formed the 7600 BP Shiveluch tephra deposit. Such phreatomagmatic eruptions in general tend to (1) be derived from basalts that ascend relatively fast, possibly driven by deep CO_2_-degassing and (2) therefore often carry abundant xenoliths.

Relatively short timescales of hundred days to a few years were documented for ascent of mafic magmas from mantle depths^5^. However, in these olivine crystals Fo-zoning had already been erased by diffusion processes and the width of the Ni-diffusion zone was about 100 μm, i.e. much wider than the width of the Ni-diffusion zone of 3–13 μm that we observe here (Table SM6-A). The time scales for ascent of magmas and olivine crystals in lavas of the Shiveluch 7600 BP eruption apparently rose to the surface by orders of magnitude faster.

Our research shows that crystal growth, mixing and diffusion processes on the way from mantle source to surface may be quite complex and the time of ascent can be fast – just a few days before the eruption.

## Methods

### Microprobe analyses

All measurements were conducted with a JEOL JXA 8900RL electron microprobe at the GZG (Geowissenschaftliches Zentrum Göttingen), Göttingen University. We used specifically designed high-precision methods based on increased current and increased voltage^56^. The electron microprobe was configured at accelerating voltage of 20 kV, beam current of 300 nA, and focused beam of 0–5 μm in diameter. The methods allowed us to analyse olivine profiles for major and trace elements, to conduct precision microelement measuring for thermobarometry, and to build elemental maps. All methods including standards and references are described in the Supplements SM1.1-SM1.2.

### Crystal orientation

The crystal orientation was determined via electron backscatter diffraction^57^ (EBSD) on a Quanta 200 F instrument at the Crystallography Department at the GZG, Göttingen University. For EBSD analysis, the thin sections were polished in addition to the normal procedure for EMS to a final polishing fineness of 0.05 μm Al_2_O_3_. Every sample was covered with a very thin carbon layer to minimize electrostatic charge due to the high vesicularity of the samples. Several points were measured along the compositional profile of every grain to ensure that crystal orientation does not change within the crystal due to cracks or deformation. All details are included in Supplement SM1.3, the results of measurements are shown in Tables SM2-B and SM2-C.

### P-T-*f*O_2_ conditions

Temperature estimates for olivine crystallization are based on Al-in-olivine thermometry^36^, which utilizes the Al-Cr distribution between olivine and spinel (Table SM3-A). Oxygen fugacity was estimated by using the improved Ballhaus-Berry-Green ol-opx-sp oxybarometer^58^. To calculate the pressure, we use H_2_O estimates in the melt as 4%^59^. Pressure was determined from cpx-melt compositions and cpx-only barometry^34^. The details of the P-T-*f*O_2_ estimates are included in Supplement SM3, mineral microprobe analyses used for P-T-*f*O_2_ estimates and individual values are shown in Tables SM3-A, SM3-B, SM3-C.

### Diffusion coefficients

Diffusion coefficients were calculated using estimated P-T-*f*O_2_ conditions with correction for crystal orientation (Table SM2-B), as described in Supplement SM3. Table SM3-C contains diffusion coefficients for all zones of the crystals, calculated^4^ with P-T-*f*O_2_ conditions and Fo content in these zones. In our calculations for every profile we selected the values of the slowest and fastest diffusion coefficients. For modelling of diffusion inside the cores we used external P-T-*f*O_2_ conditions determined for the transition zones. For modeling of diffusion in the transition zone we used P-T-*f*O_2_ conditions determined for the overgrowth.

### Analytical approximations of diffusion profiles

For the approximation of the measured profiles by the least square method in the case of outer core diffusion we used the analytical solution of the one-dimensional problem for diffusion in a semi-infinite medium (Eq. 3.13 in ref.^44^). For the initial conditions and the boundary condition at infinity we used the values of Fo and Ni in the centres of a olivine cores. For the boundary conditions of the dissolved margin of the cores we used the estimate (Fig. 5c) values Fo_dm_ = 88.7 and NiO_dm_ = 0.23 wt.%. Simultaneous adjustment for Fo and Ni solutions allowed to find the diffusion times and the position of the dissolved margin for every profile. All details are included in Supplement SM4 and Table SM4-A. An example of the approximation between data and the model is given in Fig. 6a.

For the approximation of the measured profiles by the least squares method in the case of advanced core diffusion we used the analytical solution of the spherically symmetric problem for diffusion in a sphere (Eq. 6.18 in ref.^44^). For the initial conditions we used the composition of the most magnesian core SHIV-08-05 17 Ol-7 from group 1, Fo_core_ = 92.16 and NiO_core_ = 0.48 wt.%. For the boundary condition on the margin of the sphere we used the average composition of the olivine from group 4, Fo_dm_ = 86.4 and NiO_dm_ = 0.22 wt.%. Simultaneous adjustment for Fo and Ni solutions allowed us to find the diffusion time and the position of the dissolved margin for every profile. All details are included in Supplement SM5 and Table SM5-A, an example for the model fits can be found in Fig. 6b.

For the approximation of the measured profiles by the least squares method in the case of transition zones we used the analytical solution of the one-dimensional problem for diffusion in an infinite medium (Eq. 3.13 in ref.^44^). The initial and boundary conditions on both sides of the diffusion zones were automatically determined by parameter fitting of the analytical solution. The solutions for Fo and Ni were approximated independently. All details are included in Supplement SM6 and Table SM6-A, an example for the model approximation can be found in Fig. 6c.

## Electronic supplementary material

Supplementary materials

Supplementary Table 1.1

Supplementary Table 1.2

Supplementary Table 2

Supplementary Table 3

Supplementary Table 4

Supplementary Table 5

Supplementary Table 6
