# Supplementary material for: Growth of, and diffusion in, olivine in ultra-fast ascending basalt magmas from Shiveluch volcano
Source: Sci Rep. 2018 Aug 6;8:11775. doi: 10.1038/s41598-018-30133-1 (PMC6079000; doi:10.1038/s41598-018-30133-1)
Supplement: Supplementary file 1 — Supplementary materials [file 41598_2018_30133_MOESM1_ESM.pdf]

Growth of, and diffusion in, olivine in ultra-fast ascending basalt magmas from Shiveluch volcano

Supplementary materials

Boris Gordeychik[1, 2, *], Tatiana Churikova[2, 3], Andreas Kronz[2],
Caren Sundermeyer[2], Alexander Simakin[1], and Gerhard Wörner[2]

[1] Institute of Experimental Mineralogy, Russian Academy of Sciences, Chernogolovka, 142432, Russia

[2] Geowissenschaftliches Zentrum Göttingen, Abteilung Geochemie, Universität Göttingen, Göttingen, 37077, Germany

[3] Institute of Volcanology and Seismology, Far East Branch, Russian Academy of Sciences, Petropavlovsk-Kamchatsky, 683006, Russia

[*] Correspondence and requests for materials should be addressed to B.G. (email: gordei@mail.ru)

Content

SM1. Analytical techniques 3
SM1.1. Mineral microprobe analyses 3
SM1.2. Element distribution maps 4
SM1.3. EBSD analysis 4
SM1.4. Description of the tables 4
Table SM1.1-A. EMS measurement conditions 4
Table SM1.1-B. San Carlos standards measured during olivine analysis and analytical statistics 4
Table SM1.1-C. Conditions of measurements for element maps 4
Table SM1.1-D. Element distribution maps 4
Tables SM1.2. A systematic analysis and cross-calibration of distinct batches of San Carlos olivines 4
SM2. Profile measurements 5
SM2.1. Profile SHIV-08-05 17 Ol-8-2 5
SM2.2 Description of the tables 6
Table SM2-A. Raw microprobe data on studied crystal profiles 6
Table SM2-B. Raw crystal orientation data and calculations of the geometry factor 6
Table SM2-C. Plots and images for all profiles and grains 7
SM3. Estimating P-T-*f*O2 conditions of olivine crystallization 8
SM3.1. Description of the tables 8
Table SM3-A. Aluminum-in-olivine thermometry results using olivine-spinel pairs 8
Table SM3-B. Clinopyroxene and plagioclase compositions 9
Table SM3-C. P-T-fO_2 conditions and diffusion coefficients in different crystal zones 9
SM4. Outer core diffusion times estimation 10
SM4.1. Description of the tables 12
Table SM4-A. The time intervals for the outer core diffusion 12
SM5. Advanced core diffusion times estimation 13
SM5.1. Description of the tables 16
Table SM5-A. The time intervals for advanced core diffusion 16
SM6. Core-overgrowth diffusion times estimation 17
SM6.1. Description of the tables 19
Table SM6-A. The time intervals for the diffusion in the transition zone 19
References 20

SM1. Analytical techniques

SM1.1. Mineral microprobe analyses

All mineral compositions, geochemical profiles and elemental maps measured on olivine were determined using a JEOL JXA 8900RL microprobe equipped with 5 wavelength dispersive spectrometers (WDS) installed at the GZG (Geowissenschaftliches Zentrum Göttingen) of the Georg-August Göttingen University. The program to analyse olivine compositions with high precision for trace elements was modified based on a published approach[1]. Olivine measurements were run at 20 kV accelerating voltage and a focused beam of 300 nA and 1-5 μm diameter. For standardization of major and trace elements, we used a set of synthetic and natural standards. Peak counting times for major elements were 15 s, for Mn – 60 s, for Cr, Zn and Ca – 120 s, for Co – 180 s, for Ni and Al – 260 s, and for P – 300 s (Table SM1.1-A). To ensure accuracy and precision, as well as to correct for instrumental drift we used a San Carlos olivine crystal, but not the USNM-San Carlos sample (USNM 111312/444), because of the limited availability of the USNM material. Commercially available San Carlos olivine as well as the original USNM San Carlos RM was found to be variable in composition for different crystal fragments[2] (Fo 87.5-91.5 for non-USNM San Carlos olivine; Fo 89.6-90.5 for USNM 111312/444). The "in house" crystal (SC-Goe) of 3 mm diameter was carefully checked for homogeneity and was characterized by rigorous quantitative measurements of original USNM 111312/444 crystals and other standards (Wollastonite: Si, MgO: Mg, Hematite: Fe). SC-Goe gives reliable results for stoichiometry, but differs from USNM 111312/444 significantly in FeO and NiO concentrations (Table SM1.2). For other elements, no systematic offsets within the errors by counting statistics and the uncertainties of matrix correction procedures were observed. Thus, the published reference values were used for MgO, MnO, SiO_2[3]; for Cr_2O_3[4]; for CaO[5]; for Al_2O_3, P_2O_5, CoO, ZnO[1]. SC-Goe was analysed during all measurements after 30-50 analyses of unknowns. The quality of analyses was also controlled by mineral stoichiometry for the olivine. The absolute average errors (2σ) by counting statistics for major and trace elements on the SC-Goe olivine are (mass-%): SiO_2 ~ 0.24, MgO ~ 0.3, FeO ~ 0.12 and NiO ~ 0.003, MnO ~ 0.004, CaO ~ 0.0016, Cr_2O_3 ~ 0.0022, Al_2O_3 ~ 0.0013, ZnO ~ 0.0033, CoO ~ 0.0017, and P_2O_5 ~ 0.0017. The standard deviations calculated from n=63 total measurements are 1.2- to 3-times larger than the predicted error by counting statistics, except for Al_2O_3, where the standard deviation of 63 measurements of San Carlos olivine is 6.7 times larger. Relative deviations from recommended values are better than 3% for all oxides except for Co, P (5%), Cr (9%), and Zn (~48%). Due to a non-linear behavior of the background signal near the Co-Kα-line resulting from the nearby Fe-Kβ interference, a linear correction factor was applied to correct for the systematic underestimation of the raw net-signals. Statistics for the olivine analyses are given in Table SM1.1-B. Detection limits are calculated from the error by counting statistics of the background noise and given as 2-sigma values in Table SM1.1-B.

To increase the analytical precision for aluminum in olivine for the application of the sp-ol geothermometer[6], a specially tuned analytical program for Al was designed where Al_2O_3 was simultaneously measured on two spectrometers. This technique allowed to an improvement to the 2 sigma error by counting statistics to ±8.4 $\mu g \cdot g^{-1}$ and a reduction in the detection limit down to 5.6 $\mu g \cdot g^{-1}$.

The compositions of other rock-forming minerals in basaltic tephra (spinel, pyroxene, and plagioclase) were determined using routine procedures at 15-20 kV and 15-20 nA with a 1-10 μm beam diameter. Peak counting times for major elements were 15-30 s, and for trace elements 60 s. Analytical precision was better than 5% for most major elements and 7% for K and P[7].

SM1.2. Element distribution maps

Element distribution maps of Fe, Mg, Ni, Ca, Cr, Al, and P in olivine were carried out on whole olivine grains or on some sections or individual zones of olivine crystals with excitation conditions similar to those used for the high-precision quantitative analyses – 20 kV and 300 nA. Depending on the size of the analyzed area and on the required spatial resolution, step width and dwell time per pixel were changed from 0.1 to 1.8 μm and from 100 to 210 ms, respectively. The covered areas range between 300 and 800 μm^2 and the total acquisition time range from 5 to 15 hours per map. A focused beam was used for all element maps. The full measurement conditions for 5 elemental maps can be found in Table SM.1-C.

SM1.3. EBSD analysis

Diffusion modelling in olivines requires the exact knowledge of crystal orientation due to the strong anisotropic diffusion of Fo-Ni in olivine[8-10], which can be described as $D_a = D_b = D_c/6$. Therefore, the orientation of every crystal must be measured to obtain correct diffusion times. The crystal orientation was determined via electron backscatter diffraction[11] (EBSD) on a Quanta 200 F instrument at the Crystallography Department at the GZG, Georg-August Göttingen University.

For EBSD analysis, the thin sections were polished in addition to the normal procedure for EMS to a final polishing fineness of 0.05 μm Al_2O_3. Every sample was covered with a very thin carbon layer to minimize electrostatic charge due to the high vesicularity of the samples. Several points were measured along the compositional profile of every grain to ensure that crystal orientation does not change within the crystal due to cracks or deformation.

The software OIM Analysis[12] was used to calculate the angles between the line profile and the crystallographic axes obtained from EBSD. These angles can be used to define a diffusion coefficient along the measured profile D_{Pr} using an equation[8,13], which is:

$$D_{Pr} = D_a\, cos^2\alpha + D_b cos^2\beta + D_c cos^2\gamma \qquad \textit{(Eq. SM1.1)}$$

with D_a, D_b, and D_c as diffusion coefficients along the crystallographic axes a, b, and c, and α, β, and γ as angles between the measured profile and a-, b-, and c-axis.

SM1.4. Description of the tables

Table SM1.1-A. EMS measurement conditions

Table SM1.1-B. San Carlos standards measured during olivine analysis and analytical statistics

Table SM1.1-C. Conditions of measurements for element maps

Table SM1.1-D. Element distribution maps

Tables SM1.2. A systematic analysis and cross-calibration of distinct batches of San Carlos olivines

SM2. Profile measurements

This part of supplementary materials presents results of measuring elemental concentrations and crystal orientations along the profiles in olivine grains. We measured 27 profiles across 19 carefully selected olivine grains in eight thin sections from two samples. Table SM2-A contains the raw microprobe data. Table SM2-B contains raw orientation data.

SM2.1. Profile SHIV-08-05 17 Ol-8-2

We will demonstrate the scheme of analyzing the measured compositional profiles in olivine crystals using as example profile SHIV-08-05 17 Ol-8-2. The distribution of Fo, NiO and Cr_2O_3 are shown in Fig. SM2.1a, a Fo-Ni diagram is shown in Fig. SM2.1b. Table SM2-C contains (a) data on some other elements, (b) images of crystals in SEI and COMPO modes obtained on an electron microprobe, (c) the position of profile across the crystal, and (d) the orientation of crystallographic axes for each profile analyzed.

Even though each profile has its own details, the Fo-Ni diagram below demonstrates a representative profile SHIV-08-05 17 Ol-8-2. The core of the crystal shows a unique loop with reversing Ni and Fo zones: first Ni concentration increases at decreasing Fo, then Ni decreases at increasing Fo (red arrows at Fig. SM2.1b). At the core-overgrowth boundary, the highest gradients in Fo, Ni and Cr are coupled with a distinct Ni-kink (dashed line in Fig. SM2.1a). It is important to note that points D_1 and D_2 with highest Ni and Fo values are displaced with respect to each other (Fig. SM2.1a, Fig. SM2.1b). Starting from point D_2, the crystal evolution follows a typical fractional crystallization trend towards the margin. Several chromium (and Ca, Al, P) peaks are present within the overgrowth.

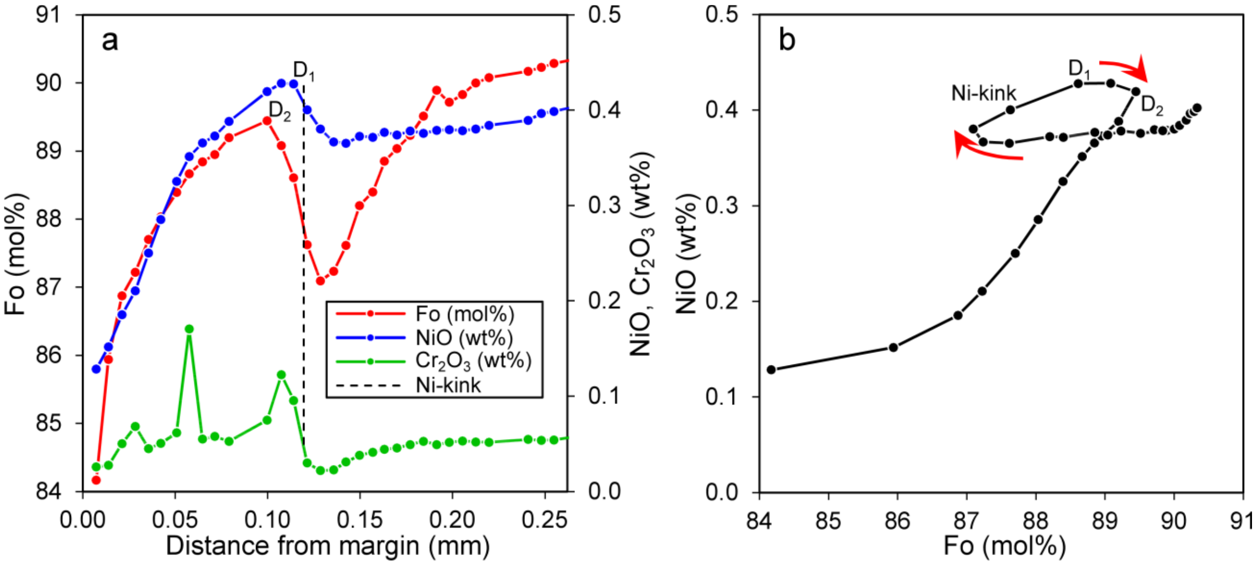

Fig. SM2.1. (a) Fo (left axis), NiO and Cr_2O_3 (the right axis) concentrations vs. distance from the crystal margin along the profile SHIV-08-05 17 Ol-8-2. Dashed line marks the position of Ni-kink. Points D1 and D2 are the maxima in NiO and Fo, respectively. (b) Diagram with loop in Fo-Ni composition. The red arrows show the multidirectional changes in olivine composition on the core-margin transect

SM2.2 Description of the tables

Table SM2-A. Raw microprobe data on studied crystal profiles

- Point – the name of measured point.
- Sample – the name of the sample SHIV-yy-nn, where SHIV means belonging of the sample to Shiveluch volcano, yy – the year of the sampling, 2 digits, nn – sequence number of sample, 2 digits.
- Thin section – the individual identification number of the thin section, usually 1-2 digits with possible presence of the dash.
- Grain, profile – the entry Ol-[g]g-[p], where Ol means the olivine grain, [g]g – sequence number of olivine grain on the current thin section, 1 or 2 digits, and p – sequence number of profile in the current grain, 1 digit. If number of profile is omitted, it means the one profile in this grain.
- Group – the number of olivine group it the terms of Fig. 2.
- Number of point – the number of point in the profile.
- x-coordinate; y-coordinate – coordinates of measured point on the thin section.
- x_S-coordinate; y_S-coordinate – coordinates of starting point of profile on the thin section.
- Distance – the distance from the current point to the starting point of the profile.
- SiO_2 … Cr_2O_3 – the measured weight concentration of oxides in olivine.
- Total – the sum of all measured oxides in olivine.
- Fo – Forsterite value – 100*(MgO/40.304)/(MgO/40.304+FeO/71.846).
- Dimensionless coordinate – the margin, Ni-kink and centres are allocated to coordinates of -1, 0, and 1, respectively. Normalized profiles are scaled linearly in both intervals inward and outward of the Ni-kink.
- r(margin) – the distance from the margin to the starting point of the profile.
- r(kink) – the distance from the Ni-kink point to the starting point of the profile.
- r(centre) – the distance from the centre of the grain to the starting point of the profile.
- r – the distance from the current point to the margin.
- xmin, ymin, xmax, ymax, scale, x-shift, y-shift, X, Y – auxiliary values for the drawing of the profiles on COMPO-images (Table SM2-C).

Table SM2-B. Raw crystal orientation data and calculations of the geometry factor

- Sample, thin section, grain, profile – see the explanation for Table SM2-A.
- Objects and values – shows the rows for the Bunge's form of three Euler angles[12]; the row for starting and ending coordinates of the profile.
- Rotation matrix – the components of rotation matrix for calculating of crystal's axes orientations.
- Axes – shows the three crystal axes rows and measured profile row.
- x and y – coordinates of stereographic projection to lower hemisphere of the three crystal axes and measured profile.
- $\cos^2$ – the squared cosines of angles between the measured profile and a-, b-, and c-axis of crystals. The "Profile" raw shows the check sum of three previous squared cosines: should be equal 1.
- Geometric factor A_{Pr}, i.e. the components of the diffusion coefficients along the a-, b- and c-axes[8] and the resulting geometric factor A_{Pr} of the diffusion coefficients along the profile.

$$A_{Pr} = cos^2\alpha + \frac{1}{6}cos^2\beta + \frac{1}{6}cos^2\gamma \qquad \textit{(Eq. SM2.1)}$$

Table SM2-C. Plots and images for all profiles and grains

- Sample, thin section, grain, profile – see the explanation for Table SM2-A
- Plots for Fo, Ni, Mn and Cr – Fo are plotted on the left axis the left axis, NiO, MnO, and Cr_2O_3 are plotted on the right axis of the plots
- Plots for Ca, Co, Al, P, Zn – CaO are plotted on the left axis, CoO, Al_2O_3, P_2O_5, and ZnO are plotted on the right axis of the plot
- SEI – images of grains from microprobe in SEI mode
- COMPO and profiles – images of grains from microprobe in COMPO mode. The positions of measured points are represented by red dots
- Projections – lower hemisphere of stereographic projection of a-, b- and c-axes of crystal

SM3. Estimating P-T-*f*O2 conditions of olivine crystallization

Because Mg, Fe, and Ni are highly diffusive elements in olivine, any temperature estimate for olivine studied here would be not correct using thermometers based on these elements. Therefore we used Al-in-olivine thermometry[6], which is based on Al-Cr distribution between olivine and spinel. Both elements have low diffusivity in both phases[14,15]. Another advantage of this method is that today it is possible to measure low concentrations of Al-in-olivine by electron microprobe at high spatial resolution and with high precision[1,4,5]. The measured data are represented in Table SM3-A. Oxygen fugacity was estimated using the approach[16] based on the improved Ballhaus-Berry-Green ol-opx-sp oxybarometer. Fugacity estimates were done on the same ol-sp pairs, which were also used for thermometry. In all cases, ol-sp pairs were measured closely at the contact of the olivine to their spinel inclusions in the different parts of the phenocrysts.

Pressure was estimated from clinopyroxene compositions and clinopyroxene-melt equilibria (Eq. 30, 31, 32b and 32c in ref.[17]). Fe-Mg diffusion in clinopyroxenes is much slower compared to olivines and even compared to orthopyroxenes[18,19] although can be complicated by strong compositional zoning. Thereby the clinopyroxene-melt barometer does not suffer from Mg-Fe diffusion. To calculate the pressure using the barometers[17], we used H_2O estimates[20] in the melt as 4%. Olivine compositions from the overgrowths ($Fo_{88-90.5}$, SM2) are in equilibrium with the whole rock (a proxy for their host melt). Six cpx grains, however, show variable compositions with Mg# 68-87 (Table SM3-B) and none was in equilibrium with the whole rock composition. However, after subtracting 5% of olivine Fo_{90} from the whole rock composition, the cpx compositions passed the equilibrium test[17]. The pressure thus determined from clinopyroxene-melt compositions was 6 to 10 kbar, consistent with the results from cpx-only barometry – 6 kbar. These data are in good agreement with previously published estimates in 7-9 kbar[21].

Temperatures estimated for the inner cores of Shiveluch olivine crystals range from 1230 to 1260 °C and are lower in outer cores (1170-1190 °C) and gradually decrease from the transition zone to the rim: 1150-1220 °C in the transition zone, 1160-1200 °C in the overgrowth and 1130-1155 °C at the rim. There are no spinel inclusions in the outermost rims, but the pre-eruption temperature determined for Shiveluch lavas of a similar composition was around 1100 °C, i.e. still slightly lower than and thus in accordance with temperatures estimated towards the rim of olivines.

Oxygen fugacities relative QFM buffer are ΔQFM=+1.04±0.26 at a pressure of 6 kbar and ΔQFM=+0.90±0.26 at 10 kbar (Table SM3-A), which is somewhat lower than previously estimated[21] for the same lavas ΔQFM=+1.8±0.15.

Table SM3-C also contains the calculation of diffusion coefficients[14,22] for all zones of the crystals for which the temperatures were determined individually. We chose the conditions for lowest and for fastest diffusion coefficients for every zone. The diffusion coefficients of Fo and Ni will be used in subsequent supplementary materials to estimate the timescales and durations of diffusive processes.

SM3.1. Description of the tables

Table SM3-A. Aluminum-in-olivine thermometry results using olivine-spinel pairs

- No. – line number.
- Position – position of measuring point in the grain's structure.
- Ol number – identification of measuring point in the olivine near spinel inclusion.
- SiO_2 … Fo – see Table SM2-A.
- Sp number – identification of measuring point in the spinel inclusion.
- MgO … ZnO – the measured weight concentration of oxides in spinel.

- Total – the sum of all measured oxides in spinel.
- Cr# – chromium number of spinel.
- T – temperature for ol-sp pair.
- Average T – Average temperature for sp inclusion in ol.
- ΔQFM – deviation from Quartz-Fayalite-Magnetite buffer.
- fO_2 – fugacity.

Table SM3-B. Clinopyroxene and plagioclase compositions

- No. – line number.
- Mineral number – identification of measuring point in the mineral.
- Mineral – type of the mineral.
- SiO_2 … NiO – the measured weight concentration of oxides in mineral.
- Mg# – Mg# for cpx.
- An# – An# for pl.
- Total – the sum of all measured oxides in mineral.

Table SM3-C. P-T-fO_2 conditions and diffusion coefficients in different crystal zones

- No. – line number.
- Position – zones of olivine.
- Av. Fo – average forsterite in zone.
- fO_2 – fugacity
- P – pressure
- T – temperature
- D_{Fo} – diffusion coefficient for forsterite.
- D_{Ni} – diffusion coefficient for nickel.

SM4. Outer core diffusion times estimation

Olivine crystals from group 1 have experienced diffusion in the outer cores. During this diffusion stage, the maximum values for Fo and NiO in the inner core remained nearly constant and only outer core parts were affected by diffusion. Such a case, ***outer core diffusion***, permits a one-dimensional model description of the diffusion process and it is possible to calculate the diffusion time from a simple analytical solution.

The inner cores of olivines from group 1 are in equilibrium with high-Mg melt and have compositions of Fo_{core}=91.8-92.1 mol. %, and NiO_{core}=0.45-0.49 wt. % (Fig. 8a, Fig. 8b). The rims of these crystals suffered diffusion at the contact to a more evolved melt in equilibrium with Fo_{dm}=88.7 mol. % and NiO_{dm}=0.23 wt. % (Fig. 5). At these parameters, there is a linear dependency between the left and right sides of the equation Eq. 3. The slope of correlation line in Fig. 5c controls the ratio D_{Ni}/D_{Fo}=0.86. In this case, the concentrations of forsterite Fo and nickel NiO along the profile will be described by following equations (see Eq. 3.13 for semi-infinite medium in ref.[23]):

$$\frac{Fo-Fo_{dm}}{Fo_{core}-Fo_{dm}} = \mathrm{Erf}\left(\frac{x-x_{dm}}{\Delta_{Fo}}\right) \qquad \textit{(Eq. SM4.1)}$$

$$\frac{NiO-NiO_{dm}}{NiO_{core}-NiO_{dm}} = \mathrm{Erf}\left(\frac{x-x_{dm}}{\Delta_{Ni}}\right) \qquad \textit{(Eq. SM4.2)}$$

$$\frac{\Delta_{Ni}}{\Delta_{Fo}} = \sqrt{\frac{D_{Ni}}{D_{Fo}}} \qquad \textit{(Eq. SM4.3)}$$

The characteristic dimensions of the diffusion zones Δ_{Ni}, Δ_{Fo} and position of x_{dm} are calculated by the least square method from equations Eq. SM4.1-SM4.3. Note that x_{dm} does not mark the margin of present crystal but the original margin of the now partially resorbed core when it was exposed to a more evolved melt. Thus, the values Δ_{Ni}, Δ_{Fo} and x_{dm} need to be evaluated from only partially preserved profiles after the resorption event.

Five profiles on three olivine grains were used for estimating the duration of outer core diffusion. The calculated values for Δ_{Ni}, Δ_{Fo} and x_{dm} are shown in Table SM4-A. The representative profiles together with locations of dissolved margins, the half-widths of the diffusion zones and the approximation curves are shown in Fig. SM4.1.

The results of analytical solutions reproduce well the measured profiles. The evaluated half-widths of the diffusion zone allows at known diffusion coefficients to calculate the diffusion time as:

$$t = \frac{\Delta_{Fo}^2}{4D_{Fo}} = \frac{\Delta_{Ni}^2}{4D_{Ni}} \qquad \textit{(Eq. SM4.4)}$$

Because the outer core diffusion occurred after the olivine crystals were moved into a more evolved melt, the P-T values specific of the transition zone (raw 3 in Table SM3-C) were used for D_{Fo} and D_{Ni} calculations. Correction for crystal anisotropy from Table SM2-B was also applied.

Contrary to our estimate for D_{Ni}/D_{Fo}=0.86 (Fig. 5c), independent data for the same temperature and compositions[14] (raw 3 in Table SM3-C) give a D_{Ni}/D_{Fo} value about 1.5. Taking into account the uncertainties in the determination of diffusion coefficients as well as the effect of P-T-fO_2, the diffusion time is estimated at 400-1800 days (last columns in Table SM4-A, and blue line in Fig. 7).

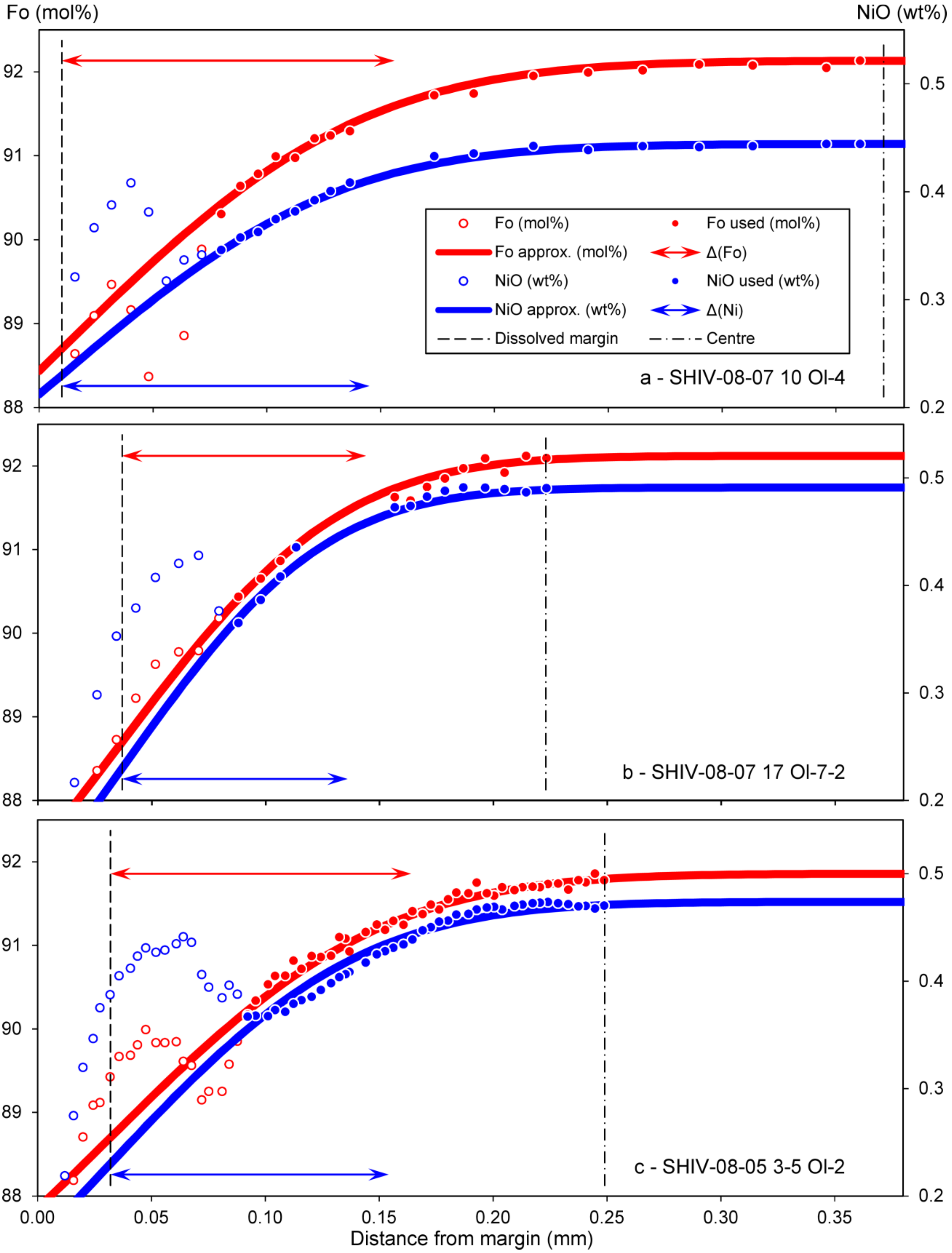

Fig. SM4.1. Three representative profiles used for estimation the time of the outer core diffusion. Forsterite profiles shown by red circles according to the axis on the left, and NiO profiles – by blue circles according the axis on the right. Only data points with filled circles were used in the calculations. Red and blue lines show the result of analytical solutions according equations Eq. SM4.1 and Eq. SM4.2 for Fo and Ni respectively. Dash-dotted lines indicate the location of the crystal centres, dotted lines are the calculated positions of the dissolved margins, red and blue arrows are characteristic dimensions of the diffusion zones Δ_{Fo} and Δ_{Ni}, respectively.

SM4.1. Description of the tables

Table SM4-A. The time intervals for the outer core diffusion

- Number – line number.
- Sample, thin section, grain, profile – see the explanation for Table SM2-A.
- Part of profile – for profiles margin-centre-margin.
- x_{dm} – distance of dissolved margin from current margin.
- x_{centre}-x_{dm} – distance of dissolved margin from the centre of grain.
- Δ_{Fo} – characteristic dimension of diffusion zone for Fo.
- Δ_{Ni} – characteristic dimension of diffusion zone for Ni.
- Geometric factor A_{pr} – see Table SM2-B.
- High D_{Fo} – the highest Fo-diffusion coefficient from line 3 of Table SM3-C.
- Low D_{Fo} – the lowest Fo-diffusion coefficient from line 3 of Table SM3-C.
- Time interval by Fo – time interval, defined by Fo-diffusion coefficients.
- High D_{Ni} – the highest Ni-diffusion coefficient from line 3 of Table SM3-C.
- Low D_{Ni} – the lowest Ni-diffusion coefficient from line 3 of Table SM3-C.
- Time interval by Ni – time interval, defined by Ni-diffusion coefficients.
- Resulting time interval – time interval, defined by Fo- and Ni-diffusion coefficients.

SM5. Advanced core diffusion times estimation

Olivine cores that were affected by diffusion from margin to centre are identified by a corresponding decrease in their maximum Fo and NiO in the core. These grains were affected by what we call ***advanced core diffusion***. To describe the diffusion effects through the entire crystal, a diffusion model should take into account the following parameters: the three-dimensional pattern of diffusion, anisotropy of the diffusion, and the complex shape of the original crystal (which is not preserved because of resorption).

A series of calculations with anisotropic crystals of variable shapes shows rather large variations in diffusion times. It is also obvious that we cannot constrain the parameters above with any confidence for the resorbed cores. For this reason our diffusion time estimation can only be an "order-of-magnitude" approximation using the simplest model where the olivine grain is represented as a sphere with radius R. In this model the olivine crystal anisotropy is controlled by the additional geometric factor A_{Pr} to the diffusion coefficient along the profile.

For example, consider the following scenario: a uniform sphere with radius R_{dm} (dm – dissolved margin, representing the original margin of the crystal that will be later dissolved) containing Fo_{core} and Ni_{core} components is moved into a melt that is in equilibrium with Fo_{dm} and Ni_{dm}. If diffusion coefficients are D_{Fo} and D_{Ni} for Fo and Ni, respectively, then for the point with coordinate r inside the sphere at time t the solution for Fo and NiO can be described as:

$$\frac{Fo - Fo_{dm}}{Fo_{core} - Fo_{dm}} = F\left(\frac{D_{Fo}t}{R_{dm}^2}, \frac{r}{R_{dm}}\right) \qquad \textit{(Eq. SM5.1)}$$

$$\frac{NiO - NiO_{dm}}{NiO_{core} - NiO_{dm}} = F\left(\frac{D_{Ni}t}{R_{dm}^2}, \frac{r}{R_{dm}}\right) \qquad \textit{(Eq. SM5.2)}$$

Here F is the solution of the diffusion equations in a sphere with constant initial conditions and given compositional values at the boundary (Eq. 6.18 in ref.[23]). It is assumed that all initial crystals have the same composition Fo_{core}=92.16 and NiO_{core}=0.48 wt. % (the composition of the most magnesian core in crystal SHIV-08-05 17 Ol-7 from group 1), and the melt around is in equilibrium with Fo_{dm}=86.4 and NiO_{dm}=0.22 wt. % (in equilibrium with olivine cores from group 4). The diffusion coefficient ratio was estimated from the diffusion trend of group 1 to group 4 core compositions (purple line in Fig. 2b) as D_{Ni}/D_{Fo}=0.74. In this case, using equations Eq. SM5.1 and Eq. SM5.2 by the least square method for each profile allows to calculate the values of R_{dm} – olivine radius before its melting, and t – time of diffusion.

Because the advanced core diffusion (as in case with outer core diffusion) occurred after the olivines were moved to more evolved melt, the P-T values characteristic of the transition zone (raw 3 in Table SM3-C) were used for D_{Fo} and D_{Ni} calculations. Correction for crystal anisotropy from Table SM2-B was also applied.

18 profiles on 15 olivine grains were used to estimate the duration of advanced core diffusion. R_{dm} and t were calculated from the measured profiles by the least square method (Table SM5-A). Representative profiles together with locations of dissolved margins and the approximation curves are shown in Fig. SM5.1.

The calculated coordinates of dissolved margins show some variation for small crystals (Fig. SM5.2). Larger crystals show consistent results, suggesting that 0.07-0.09 mm of the original crystals were dissolved. Coordinates of the dissolved margins, calculated by the outer core diffusion model (red dots on the Fig. SM5.2) and by the advanced core diffusion model (red line on the Fig. SM5.2), are close each other. This supports the validity of our assumptions and models.

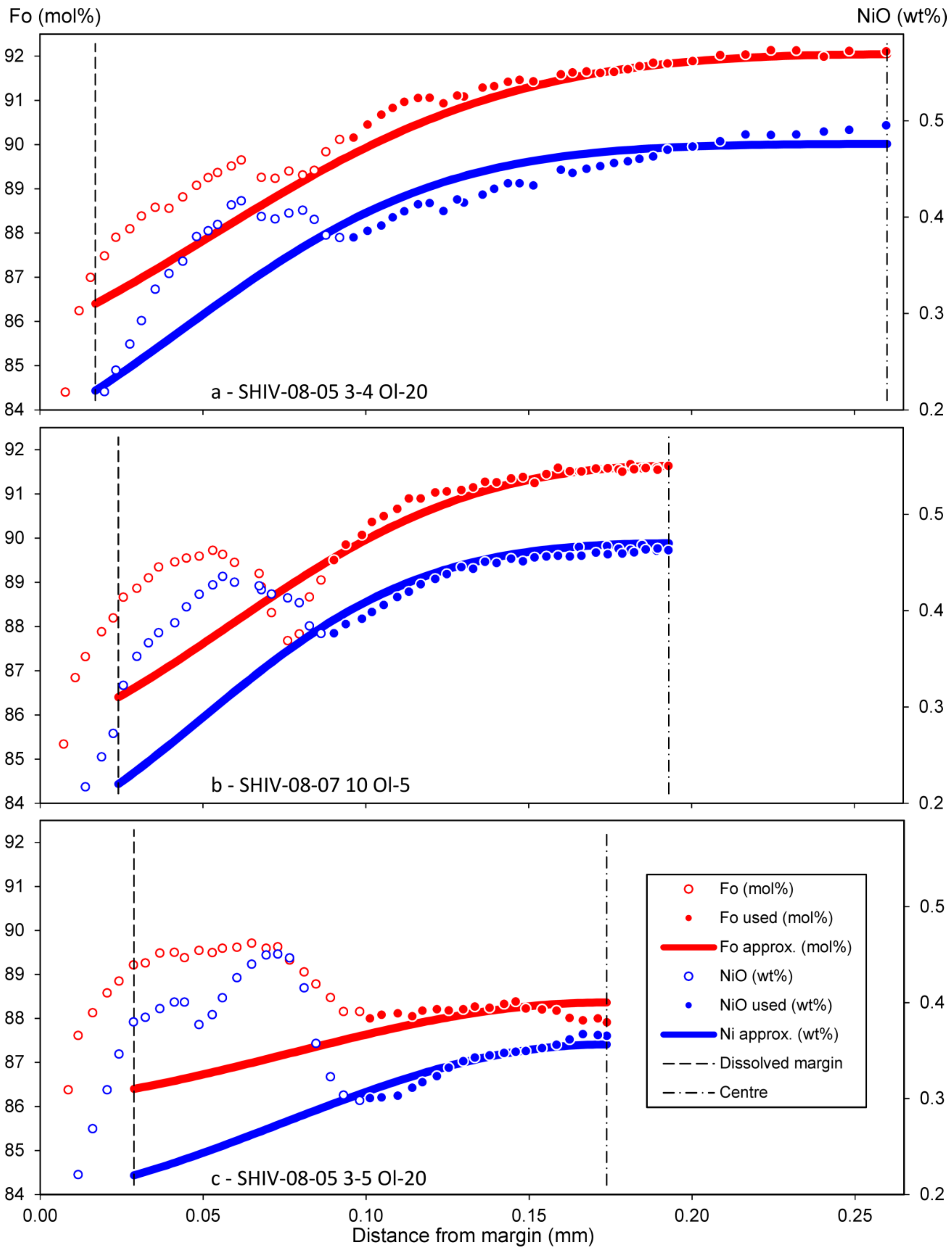

Fig. SM5.1. Three representative profiles used for estimation time of the advanced core diffusion. Forsterite (red circles) refer to the left axis, and NiO profiles (blue circles) to right axis. Only filled circles were used in the model calculations. Red and blue lines show the result of analytical solutions according equations Eq. SM6.1 and Eq. SM6.2. Dash-dotted lines indicate the location of crystal centre; dotted lines are the calculated locations of the original margins of the crystal that was later dissolved.

Our estimate of D_{Ni}/D_{Fo}=0.74 differs from published data[14] for olivines of the same composition and conditions of formation (raw 3 in Table SM3-C) which indicate a D_{Ni}/D_{Fo} ratio of about 1.5, (SM4). Taking into account the uncertainties in the definition of diffusion coefficients as well as P-T-fO_2 conditions, the diffusion time interval is in the range of 100 to 2000 days (last columns in Table SM5-A, and green line in Fig. 7).

Outer core diffusion is observed only in larger crystals (Fig. SM5.2), which had no time to diffuse into the core during the estimated diffusion time. In fact the outer core diffusion is only the special case of diffusion for large crystals whereas smaller crystals have partly or completely equilibrated during the same diffusion time. This explains nicely the close agreement between outer core diffusion times (blue line in Fig. 7) and advanced core diffusion times (green line in Fig. 7), estimated by two methods. Some discrepancies may be related to differences in Fo_{dm}, Ni_{dm} and ratio D_{Ni}/D_{Fo} estimates.

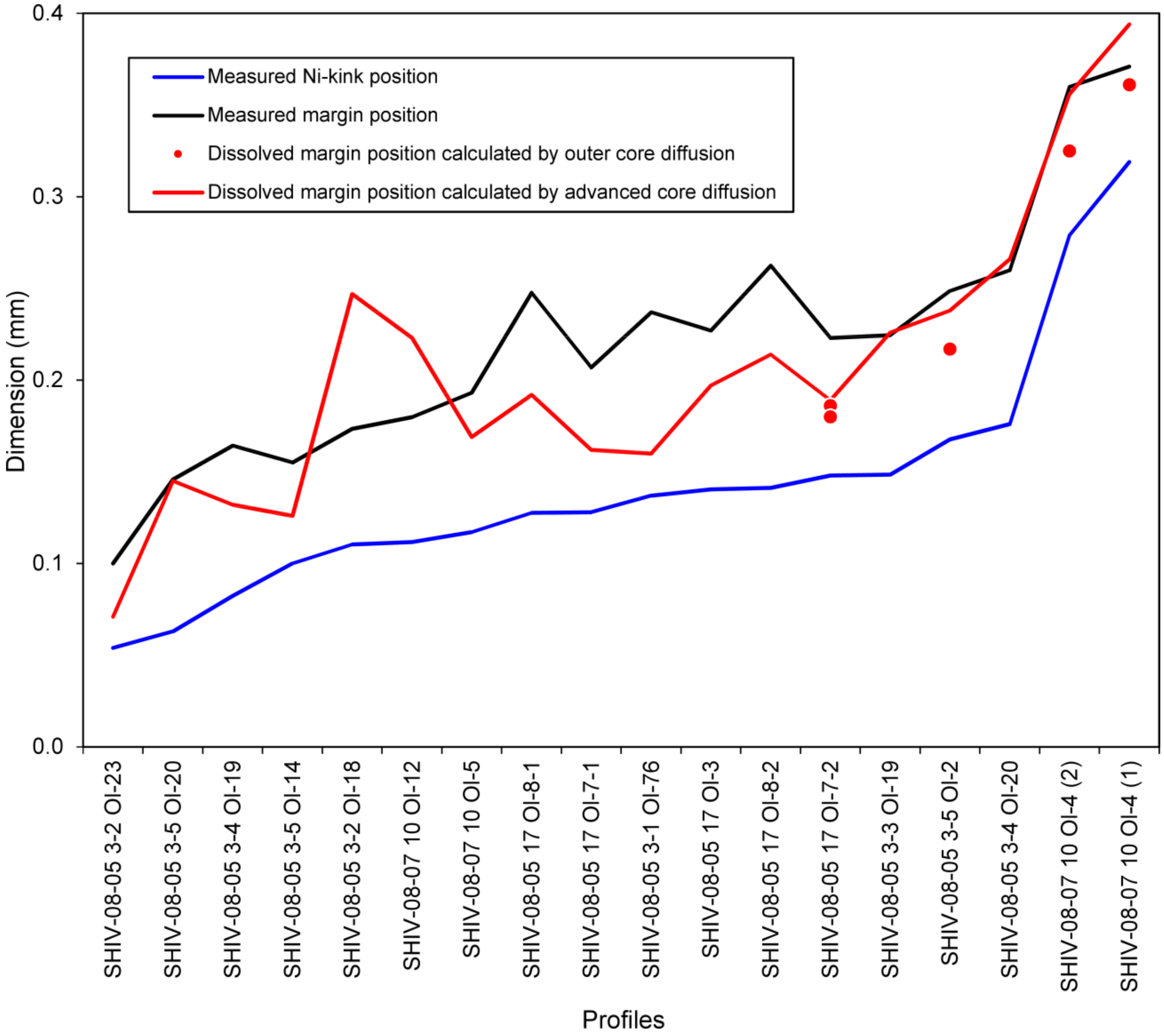

Fig. SM5.2. Measured and calculated sizes of the crystals and their zones. The crystals are sorted by their present core size, which is determined by the distance from the centre to the Ni-kink point (blue line). The crystal size is determined by the distance from the centre to margin (black line). The red line represents the coordinates of the margins of the cores before the crystals were dissolved in hot melt, calculated by advanced core diffusion. The red circles are shown for comparison and represent the coordinates of the same dissolved margins, calculated from the outer core diffusion.

SM5.1. Description of the tables

Table SM5-A. The time intervals for advanced core diffusion

- Number – line number.
- Sample, thin section, grain, profile – see the explanation for Table SM2-A.
- Part of profile – see the explanation for Table SM4-A.
- R_{dm} – distance of dissolved margin from the centre.
- Margin – distance of margin from the centre.
- Ni-kink – distance of Ni-kink from the centre.
- $D_{Fo}t$ – approximation value.
- Geometric factor A_{pr} – see Table SM2-B.
- High D_{Fo} – the highest Fo-diffusion coefficient from line 3 of Table SM3-C.
- Low D_{Fo} – the lowest Fo-diffusion coefficient from line 3 of Table SM3-C.
- Time interval by Fo – time interval, defined by Fo-diffusion coefficients.
- High D_{Ni} – the highest Ni-diffusion coefficient from line 3 of Table SM3-C.
- Low D_{Ni} – the lowest Ni-diffusion coefficient from line 3 of Table SM3-C.
- Time interval by Ni – time interval, defined by Ni-diffusion coefficients.
- Resulting time interval – time interval, defined by Fo- and Ni-diffusion coefficients.

SM6. Core-overgrowth diffusion times estimation

The transition zone with drastic changes of Fo and Ni occurs between the core and the overgrowth of the crystal. Since the transition zone was affected by diffusion, the size of the transition zone for NiO depends on the coefficient D_{Ni} and the size of the transition zone for Fo depends on the coefficient D_{Fo}, and in both cases the size is determined by the action time of diffusion. Thus, the width of the diffusion zone at the core-overgrowth boundary allows us to determine the time elapsed between the overgrowth formation and the crystal quenching after eruption to the surface. Here we apply an analytical solution of the diffusion equation for times estimations through Fo and Ni distributions across the transition zones in group 4 olivines.

The transition zone of the olivine crystals from group 4 represent a convenient case for the evaluation of the diffusion time in the system. The cores of these olivines come to equilibrium and have flat $Fo_{86\text{-}88}$, and NiO=0.2-0.3 wt. % distributions (Fig. 3a, Fig. 8b). After mixing with hot magnesian melt the olivines were partly dissolved (Fig. 8c) and then overgrown by high-Mg high-Ni olivine with $Fo_{89.5\text{-}90}$ and NiO=0.4-0.45 wt. % (Fig. 8d). Diffusion of Fo and Ni across this boundary forms the transition zone (Fig. 8e).

To model the diffusion profile through the transition zone all measured points of the core were considered because for group 4 olivines the Fo and NiO values inside the core are nearly constant (filled circles in Fig. SM6.1). However, on the outer side of the transition zone the points with lower values of Fo and NiO were excluded (open circles in Fig. SM6.1). For the ideal case when on both sides of the transition zone the values Fo and NiO would be constant at zero time, the distribution of both compositional parameters along the profile would be (see Eq. 3.13 for semi-infinite medium in ref.[23]):

$$Fo = \frac{Fo_1+Fo_2}{2} + \frac{Fo_2-Fo_1}{2} Erf\left(\frac{x-x_0^{Fo}}{\Delta_{Fo}}\right) \qquad \textit{(Eq. SM6.1)}$$

$$NiO = \frac{NiO_1+NiO_2}{2} + \frac{NiO_2-NiO_1}{2} Erf\left(\frac{x-x_0^{Ni}}{\Delta_{Ni}}\right) \qquad \textit{(Eq. SM6.2)}$$

where subscripts 1 and 2 correspond to parameters at both sides of the transition zone, Erf – error function, x_0 – initial location of the contact boundary, Δ_{Fo} and Δ_{Ni} – the characteristic half-dimension of the diffusion zone for Fo and Ni, respectively. Unknown parameters in Eq. SM6.1 and Eq. SM6.2 were calculated by the least square method that requires the solution of a system of nonlinear equations. The position of the diffusion interface, i.e. the contact boundary between the resorbed core and the overgrowth, must be the same for Fo and Ni. However, it is possible to determine this parameter for Fo and Ni separately, and after comparing them with each other, to control the quality of the model. Thus, for each Fo and Ni profile 4 parameters were determined: the compositional value to the left, the value to the right, the position of diffusion boundary and the characteristic half- dimension of the diffusion zone. The evaluated half-dimensions of the diffusion zone are related to the diffusion time as:

$$t = \frac{\Delta_{Fo}^2}{4D_{Fo}} = \frac{\Delta_{Ni}^2}{4D_{Ni}} \qquad \textit{(Eq. SM6.3)}$$

were D_{Fo} and D_{Ni} are diffusion coefficients. Because diffusion in the transition zone occurred after the overgrowth was arise on olivine core, the P-T-fO_2 values characteristic of the overgrowth (raw 4 of Table SM3-C) were used for D calculations. Correction for crystal anisotropy from Table SM2-B was also applied. The ratio of half-dimension of the diffusion zones is defined by the ratio of D_{Fo} and D_{Ni}:

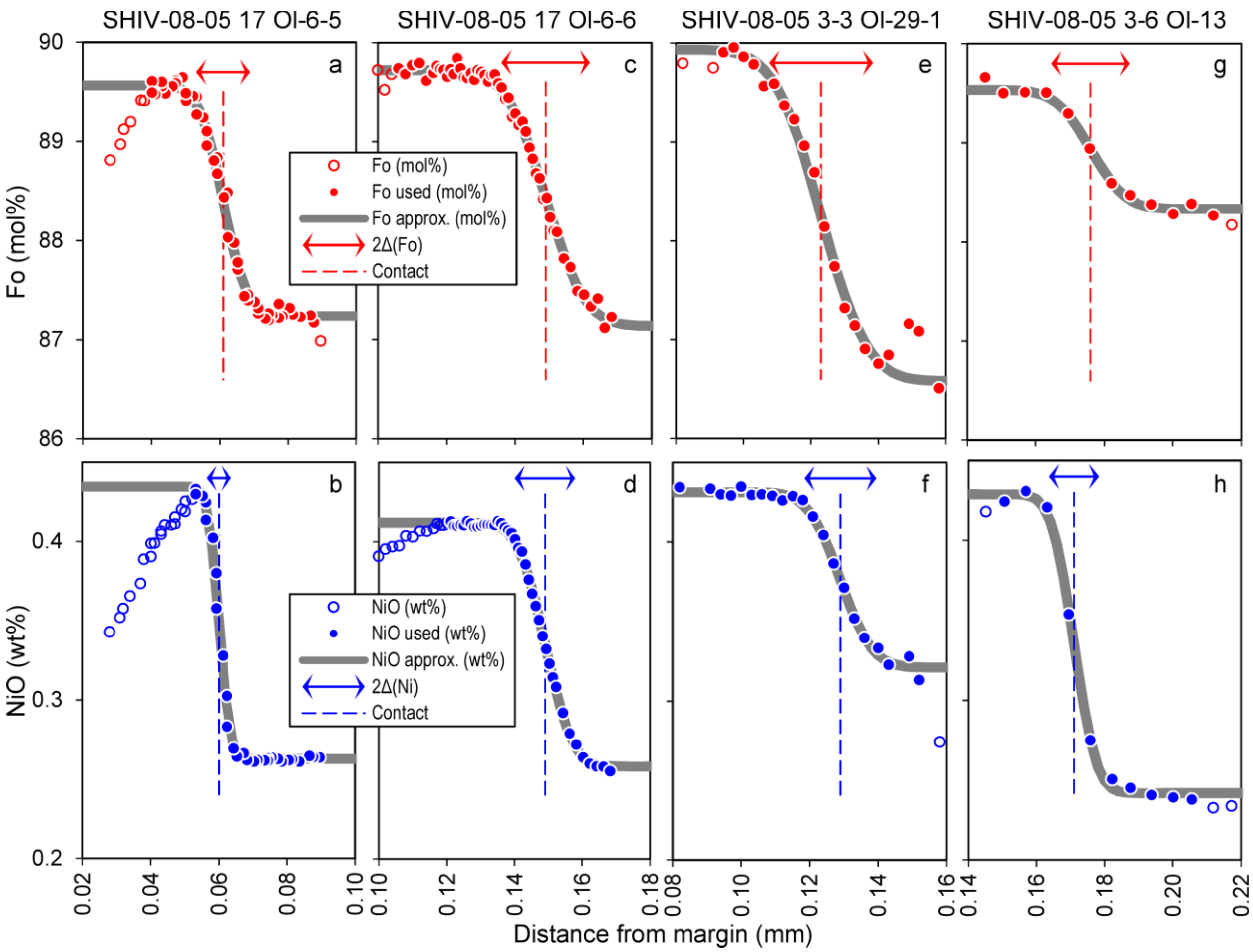

Fig. SM6.1. Four representative Fo (a, c, e, g) and Ni (b, d, f, h) profiles, plotted with the same horizontal scale. Circles – measured data. Filled circles were used in calculations, open circles were excluded. Analytical solutions using Eq. SM6.1 and Eq. SM6.2 are shown by grey lines. Dashed line – initial positions of the contact boundary. Arrows – width of the diffusion zones.

$$\frac{\Delta_{Ni}}{\Delta_{Fo}} = \sqrt{\frac{D_{Ni}}{D_{Fo}}} \qquad \textit{(Eq. SM6.4)}$$

Nine olivine profiles (all from group 4) were used for diffusion time estimates. All relevant parameters for modeling these profiles shown in Table SM6-A. Four representative profiles are shown in Fig. SM6.1.

The analytical solution reproduces well all measured profiles. The positions of the diffusion boundaries are defined for Fo and Ni and coincide well: for six definitions the difference was less than 0.001 mm, 3 definitions coincided within a range 0.005-0.007 mm. Fig. SM6.1 and Table SM6-A show that the dimension of the diffusion zone for Fo is always larger than the dimension of the diffusion zone for Ni, (8 definitions of 9). On average, the ratio (width of the diffusion zone for Ni relative to the width of the diffusion zone for Fo) is 0.6. Using this data and the equation Eq. SM6.4, the D_{Ni}/D_{Fo} ratio is estimated at about 0.4.

We simultaneously measure and model diffusion of Fo and NiO across a diffusion zone inside olivine crystals. This allows, for the first time, to directly assess their relative diffusion coefficients. In previous studies, such diffusion modeling was done separately either for Fo (e.g.[24]) or for NiO (e.g.[25]). Modeling of olivine profiles with simultaneous exchange Fo and NiO previously were described only for the rims of crystals. However, as we show in this study, such rims maybe

affected by both diffusion and crystal growth, and comparing results for Ni and Fo may not be advised in this case.

The model curves depend strongly on the relative diffusivities of Ni and Mg-Fe. We found the best fit between observations and model for $D_{Ni}<D_{Fo}$. This is in conflict with published[14] the diffusion coefficients that indicate $D_{Ni}>D_{Fo}$ However, it is known that at high P-T conditions the "*Fe-Mg interdiffusion, Ni diffusion and Mn diffusion are almost identical within the uncertainties*"[26]. We thus argue that Fo and Ni diffusion coefficients are not well enough constrained for the P-T conditions relevant here and that the apparent contradiction is within the uncertainty.

Despite our estimate of D_{Ni}/D_{Fo}=0.4 for core-overgrowth diffusion, the approximation[14] for the same conditions (raw 3 in Table SM3-C) give a D_{Ni}/D_{Fo} ratio about 1.6. Taking into account the uncertainties in the definition of diffusion coefficients as well as P-T-fO_2 conditions, the diffusion time interval is 1-10 days (last columns in Table SM6-A, and red line in Fig. 7).

SM6.1. Description of the tables

Table SM6-A. The time intervals for the diffusion in the transition zone

- Number – line number.
- Sample, thin section, grain, profile – see the explanation for Table SM2-A.
- Part of profile – see the explanation for Table SM4-A.
- Fo_1, Fo_2, Δ_{Fo}, x_0(Fo) – parameters, defined for Fo-approximation Eq. SM6.1.
- NiO_1, NiO_2, Δ_{Ni}, x_0(Ni) – parameters, defined for Ni-approximation Eq. SM6.2.
- x_0(Fo)-x_0(Ni) – differences in calculations of contact positions for Fo and for Ni.
- Geometric factor A_{pr} – see Table SM2-B.
- High D_{Fo} – the highest Fo-diffusion coefficient from line 4 of Table SM3-C.
- Low D_{Fo} – the lowest Fo-diffusion coefficient from line 4 of Table SM3-C.
- Time interval by Fo – time interval, defined by Fo-diffusion coefficients.
- High D_{Ni} – the highest Ni-diffusion coefficient from line 4 of Table SM3-C.
- Low D_{Ni} – the lowest Ni-diffusion coefficient from line 4 of Table SM3-C.
- Time interval by Ni – time interval, defined by Ni-diffusion coefficients.
- Resulting time interval – time interval, defined by Fo- and Ni-diffusion coefficients.

References

1. Batanova, V. G., Sobolev, A. V. & Kuzmin, D. V. Trace element analysis of olivine: High precision analytical method for JEOL JXA-8230 electron probe microanalyser. *Chem. Geol.* **419**, 149-157, doi:http://doi.org/10.1016/j.chemgeo.2015.10.042 (2015).

2. Fournelle, J. H. An investigation of “San carlos olivine”: Comparing USNM-distributed material with commercially available material. *Microscopy and Microanalysis* **17**, 842-843, doi:http://doi.org/10.1017/S1431927611005083 (2011).

3. Jarosewich, E., Nelen, J. A. & Norberg, J. A. Reference samples for electron microprobe analysis. *Geostandards Newsletter* **4**, 43-47, doi:http://doi.org/10.1111/j.1751-908X.1980.tb00273.x (1980).

4. Spandler, C. & O’Neill, H. S. C. Diffusion and partition coefficients of minor and trace elements in San Carlos olivine at 1,300°C with some geochemical implications. *Contributions Mineral. Petrol.* **159**, 791-818, doi:http://doi.org/10.1007/s00410-009-0456-8 (2010).

5. Sobolev, A. V., Hofmann, A. W., Kuzmin, D. V., Yaxley, G. M., Arndt, N. T., Chung, S.-L., Danyushevsky, L. V., Elliott, T., Frey, F. A., Garcia, M. O., Gurenko, A. A., Kamenetsky, V. S., Kerr, A. C., Krivolutskaya, N. A., Matvienkov, V. V., Nikogosian, I. K., Rocholl, A., Sigurdsson, I. A., Sushchevskaya, N. M. & Teklay, M. The amount of recycled crust in sources of mantle-derived melts. *Science* **316**, 412-417, doi:http://doi.org/10.1126/science.%201138113 (2007).

6. Coogan, L. A., Saunders, A. D. & Wilson, R. N. Aluminum-in-olivine thermometry of primitive basalts: Evidence of an anomalously hot mantle source for large igneous provinces. *Chem. Geol.* **368**, 1-10, doi:http://doi.org/10.1016/j.chemgeo.2014.01.004 (2014).

7. Ginibre, C., Kronz, A. & Wörner, G. High-resolution quantitative imaging of plagioclase composition using accumulated backscattered electron images: new constraints on oscillatory zoning. *Contributions Mineral. Petrol.* **142**, 436-448, doi:http://doi.org/10.1007/s004100100298 (2002).

8. Costa, F. & Chakraborty, S. Decadal time gaps between mafic intrusion and silicic eruption obtained from chemical zoning patterns in olivine. *Earth Planet. Sci. Lett.* **227**, 517-530, doi:http://doi.org/10.1016/j.epsl.2004.08.011 (2004).

9. Dohmen, R., Becker, H.-W. & Chakraborty, S. Fe–Mg diffusion in olivine I: experimental determination between 700 and 1,200°C as a function of composition, crystal orientation and oxygen fugacity. *Phys. Chem. Miner.* **34**, 389-407, doi:http://doi.org/10.1007/s00269-007-0157-7 (2007).

10. Dohmen, R. & Chakraborty, S. Fe–Mg diffusion in olivine II: point defect chemistry, change of diffusion mechanisms and a model for calculation of diffusion coefficients in natural olivine. *Phys. Chem. Miner.* **34**, 409-430, doi:http://doi.org/10.1007/s00269-007-0158-6 (2007).

11. Prior, D. J., Boyle, A. P., Brenker, F., Cheadle, M. C., Day, A., Lopez, G., Peruzzi, L., Potts, G., Reddy, S., Spiess, R., Timms, N. E., Trimby, P., Wheeler, J. & Zetterstrom, L. The application of electron backscatter diffraction and orientation contrast imaging in the SEM to textural problems in rocks. *Am. Mineral.* **84**, 1741-1759, doi:http://doi.org/10.2138/am-1999-11-1204 (1999).

12. *Welcome to OIM Data Collection: OIM DC 7.2 Manual* (EDAX, 2013).

13. Philibert, J. *Atom Movements: Diffusion and Mass Transport in Solids* (EDP Sciences, 1991).

14. Chakraborty, S. Diffusion coefficients in olivine, wadsleyite and ringwoodite. *Rev. Mineral. Geochem.* **72**, 603-639, doi:http://doi.org/10.2138/rmg.2010.72.13 (2010).

15. Suzuki, A. M., Yasuda, A. & Ozawa, K. Cr and Al diffusion in chromite spinel: experimental determination and its implication for diffusion creep. *Phys. Chem. Miner.* **35**, 433, doi:http://doi.org/10.1007/s00269-008-0238-2 (2008).

16. Nikolaev, G. S., Ariskin, A. A., Barmina, G. S., Nazarov, M. A. & Almeev, R. R. Test of the Ballhaus-Berry-Green Ol-Opx-Sp oxybarometer and calibration of a new equation for estimating the redox state of melts saturated with olivine and spinel. *Geochem. Int.* **54**, 301-320, doi:http://doi.org/10.1134/s0016702916040078 (2016).

17. Putirka, K. D. Thermometers and barometers for volcanic systems. *Rev. Mineral. Geochem.* **69**, 61-120, doi:http://doi.org/10.2138/rmg.2008.69.3 (2008).

18. Müller, T., Dohmen, R., Becker, H. W., ter Heege, J. H. & Chakraborty, S. Fe–Mg interdiffusion rates in clinopyroxene: experimental data and implications for Fe–Mg exchange geothermometers. *Contributions Mineral. Petrol.* **166**, 1563-1576, doi:http://doi.org/10.1007/s00410-013-0941-y (2013).

19. Cherniak, D. J. & Dimanov, A. Diffusion in pyroxene, mica and amphibole. *Rev. Mineral. Geochem.* **72**, 641-690, doi:http://doi.org/10.2138/rmg.2010.72.14 (2010).

20. Portnyagin, M., Bindeman, I., Hoernle, K. & Hauff, F. Geochemistry of primitive lavas of the Central Kamchatka Depression: magma generation at the edge of the Pacific plate in *Volcanism and Subduction: The Kamchatka Region* Vol. 172 *Geophysical Monograph Series* (eds Eichelberger, J. *et al.*) 199-239 (AGU, 2007).

21. Gorbach, N. V. & Portnyagin, M. V. Geology and petrology of the lava complex of Young Shiveluch Volcano, Kamchatka. *Petrology* **19**, 134-166, doi:http://doi.org/10.1134/s0869591111020068 (2011).

22. Girona, T. & Costa, F. DIPRA: A user-friendly program to model multi-element diffusion in olivine with applications to timescales of magmatic processes. *Geochem. Geophys. Geosys.* **14**, 422-431, doi:http://doi.org/10.1029/2012GC004427 (2013).

23. Crank, J. *The Mathematics of Diffusion* (Oxford University Press, 1975).

24. Kahl, M., Chakraborty, S., Pompilio, M. & Costa, F. Constraints on the nature and evolution of the magma plumbing system of Mt. Etna volcano (1991–2008) from a combined thermodynamic and kinetic modelling of the compositional record of minerals. *J. Petrol.* **56**, 2025-2068, doi:http://doi.org/10.1093/petrology/egv063 (2015).

25. Ruprecht, P. & Plank, T. Feeding andesitic eruptions with a high-speed connection from the mantle. *Nature* **500**, 68-72, doi:http://doi.org/10.1038/nature12342 (2013).

26. Holzapfel, C., Chakraborty, S., Rubie, D. C. & Frost, D. J. Effect of pressure on Fe–Mg, Ni and Mn diffusion in (FexMg1−x)2SiO4 olivine. *Phys. Earth Planet. Inter.* **162**, 186-198, doi:http://doi.org/10.1016/j.pepi.2007.04.009 (2007).
